# S-Adenosylmethionine Affects Cell Cycle Pathways and Suppresses Proliferation in Liver Cells

**DOI:** 10.7150/jca.25422

**Published:** 2019-07-22

**Authors:** Lu Yan, Xujun Liang, Huichao Huang, Guiying Zhang, Ting Liu, Jiayi Zhang, Zhuchu Chen, Zhuohua Zhang, Yongheng Chen

**Affiliations:** 1 Institute of Precision Medicine, The Xiangya Hospital, State Key Laboratory of Medical Genetics, Xiangya Medical School, Central South University, Changsha, Hunan 410078, China; 2 NHC Key Laboratory of Cancer Proteomics & Laboratory of Structural Biology, Xiangya Hospital, Central South University, Changsha, Hunan 410008, China; 3 Department of Gastroenterology, The Xiangya Hospital, Central South University, Changsha 410008, China

**Keywords:** S-Adenosylmethionine, Liver cell, Cell cycle, Proliferation

## Abstract

S-Adenosylmethionine (SAMe) is a kind of common liver-protection medicine. Recent studies have shown that SAMe has the inhibitory effects on hepatocellular carcinoma (HCC). But the specific mechanism has not been elucidated. Here, we examine the effects and relevant mechanisms of SAMe on human hepatocellular carcinoma cell HepG2 and mouse hepatocyte AML12. We applied the technique of RNA sequencing (RNA-Seq) to identify the differentially expressed genes between HepG2 cells which were treated with SAMe or not. And western blot and Quantitative RT-PCR was used to confirm some of these genes. To investigate the response to SAMe treatment, cell proliferation assay (MTS) and flow cytometry-based assays were carried out. A total of 472 SAMe-related genes were identified by RNA-Seq. We found that differentially expressed genes were enriched in cell cycle related signaling pathway significantly by the KEGG and GO Pathway enrichment analysis. Through the construction of protein-protein interaction network, we observed the module associated with cell cycle is in the core of the whole network. All these results implied that cell cycle pathway may be very important in the regulation of SAMe effected on HepG2 cells. Then the RNA-Seq-characterized genes involved in cell cycle (MCM3, MCM4, and E2F1) were confirmed by Western blot and Quantitative RT-PCR in HepG2 and AML12 cells. MTS analysis showed that SAMe could diminish cell proliferation. And flow cytometry-based assays indicated that treatment with SAMe altered cell cycle kinetic S phase cell cycle arrest. Altogether, our data uncovered the evidence of the antiproliferative action of SAMe in liver cells, and SAMe could lead to cell cycle inhibition by up-regulating MCM3, MCM4 and E2F1 expression. It provided an important theoretical basis for the clinical chemoprevention and treatment in HCC of SAMe.

## Introduction

Hepatocellular carcinoma (HCC) is one of the most common malignancy worldwide and the second leading global cause of death related to cancer, with over 600,000 people affected 1,2,3. Although increased focus and research, the clinical treatment of HCC remains a challenge. Only about 15% of patients with early-stage HCC could accept treatments such as hepatic resection and liver transplant which offer relatively good prognosis, but the majority of patients are diagnosed with advanced-stage HCC 4,5,6. Conventional chemotherapy which is lacking in sensitivity and specificity is rarely used to treat HCC because of its serious toxicity and minimal success rate of 10%-20% 7,8. One of the most important reasons for the poor prognosis is the lack of curative therapies, especially in the advanced stage. Thus, novel therapeutic methods and agents to HCC are extremely urgent needed.

S-Adenosylmethionine (SAMe) is a naturally occurring compound found in all living cells 9. And it is a common biological methyl donor involved in methyl group transfer reactions and the intermediary metabolite related to the synthesis of glutathione and cysteine in the liver 10,11. Chronic liver disease could result declining SAMe levels and hepatic SAMe deficiency is connected with increased oxidative stress^12^, abnormal lipid homeostasis^13^ and genomic instability^14^. As a kind of common liver-protection medicine, SAMe treatment is well established not only to protect against hepatic acute injury in clinic practice, but also to decrease fibrosis in multiple experimental models 15,16. And no significant adverse events have been reported by using SAMe in clinical trials so far 17. In the last few years, several researches have focused on the mechanism of SAMe as a key regulator in different tumors. It has been reported that proliferative response could be inhibited by SAMe in breast and colon cancer cells 18,19,20. Ilisso CP and his colleagues found that SAMe could induce apoptosis in osteosarcoma cells by affecting ERK1/2 and Stat3 pathways 21. SAMe could reduce expression of MAFG in response to cholestasis, and the expression of MAFG increases in human cholangiocarcinoma and HCC specimens 22. And SAMe is pro-apoptotic in HCC by inducing Bcl-xs expression selectively but not in normal hepatocytes 23. SAMe also exhibited anti-angiogenic properties 24. Although studies have shown that SAMe has the inhibitory effects on HCC, but the specific mechanism has not been well elucidated.

As the incidence of hepatocellular carcinoma is to accelerate worldwide, chemoprevention which has received little attention must to be an important area 25. Given SAMe's excellent safety property, it is very necessary to further clarify the mechanisms and efficacy of SAMe in preventing HCC. In this report, the differentially expressed genes between HepG2 cells which were treated with SAMe or not were identified by the technique of RNA sequencing (RNA-Seq). The effects and relevant mechanisms of SAMe on human hepatocellular carcinoma cell HepG2 and mouse hepatocyte AML12 was also analyzed. The aim was to increased knowledge on the treatment of HCC with SAMe.

## Materials and methods

### Cell lines and culture

Our lab maintained the Human hepatocellular carcinoma HepG2 cells. The cells were cultured in DMEM (Gibco) supplemented with fetal bovine serum (10%, FBS, Gibco). Mouse hepatocyte AML12 cells were kindly provided by Stem Cell Bank, Chinese Academy of Sciences. Here, we used a murine (AML12) rather than a human liver cell line in our experiments is a limitation of the present study. But AML12 is a well-accepted normal liver cell line in the field of hepatology 26,27. AML12 cells were cultured in DMEM/F-12 (Gibco) with ITS Liquid Media Supplement (1%, Sigma), Dexamethasone (40ng/mL, Sigma) and fetal bovine serum (10%, FBS, Gibco). We cultured the cells at 37 ℃ with 5% CO2.

### Cell treatments

SAMe (Sigma) was prepared as 100mM solutions in ddH_2_O, and then diluted into normal growth media for cell treatments. HepG2 cells were treated with SAMe (0.5mM) for 6 hours before extracted total RNA for RNA sequencing. Different doses of SAMe (0mM, 0.5mM, 1.0mM and 2.0mM) were used to incubated with HepG2 and AML12 cells for 24 hours respectively. After treatment, trypsinization was used to harvest the cells. These cells were used in subsequent experiments.

### Identify differential expressed genes by RNA sequencing

TRIzol reagent (Invitrogen) was used to extract the total RNA according to the manufacturer's protocol. We used Qubit Fluorometer (Life Technologies) to quantify the RNA concentrations. Then we evaluated the integrity of the RNA by using Agilent Bioanalyzer 2100 (Agilent Technologies), and the samples with RNA integrity number (RIN)≥7.0 were used for sequencing library construction. Library construction was operated refer to Illumina manufacturer suggestions. Samples were run on a Hiseq^TM^ 2000 platform (Illumina) in duplicate. Before mapping the sequencing reads with poly A, adapters and low quality were pre-filtered. Filtered reads were aligned to the hg19 genome using default parameters from TopHat2 28. Differentially expressed genes were annotated and analyzed by R package DESeq 29. And the significant genes were chosen with padj<0.05.

### Enrichment

The KEGG pathway 30 annotation was conducted by KOBAS (2.0). GOseq was applied to conduct the gene ontology enrichment analysis (GO, http://www.geneontology.org/) 31. The enrichment was call differentially enriched if they exhibited a Corrected P-value<0.05. In a final step, we retrieved the interacting genes using STRING database (http://www.string-db.org/). And then, a network analysis was performed using Cytoscape software version 3.0.

### Quantitative RT-PCR analysis

To verify the RNA-Seq results, Quantitative RT-PCR analysis was used. The samples used for Quantitative RT-PCR assays were those which were used for RNA-Seq experiments. Reverse transcription was then operated using 1μg RNA and the GoScript^TM^ Reverse Transcription System (Promega). Quantitative RT-PCR analysis was done on a GoTaq® qPCR Master Mix (Promega), according to manufacturer's specification. We used the following primers:

β-Acting was used as an endogenous control. Experiments were repeated three times and operated in triplicate.

### Western blot analysis

We washed the cells using ice-cold PBS and lysed in cell lysis buffer (DingGuo, BeiJing) to extract whole-cell protein. The supernatants of cell lysates were collected after cleared by centrifugation (12,000×g for 15 min, 4 ℃). We determined the protein concentrations by BCA protein assay (DingGuo, BeiJing). 15% SDS-PAGE was used to analyzed the protein samples with 40μg, and the samples were transferred electrophoretically to PVDF film (Millipore). Specific antibodies were probed with the blots and the immunoreactive proteins were detected by an ECL kit (Millipore). The antibodies used to carry out the western blot were as followed: Rabbit anti-MCM3 antibody (Proteintech), Rabbit anti-MCM4 antibody (Proteintech), Rabbit anti-E2F1 antibody (Proteintech), Rabbit anti-β-Acting antibody (CWBIO), Peroxidase-conjugated affinipure goat anti-rabbit IgG (ZSGB-BIO). β-Acting was used as an endogenous control and experiments were repeated three times.

### Flow cytometry analysis

Cells were seeded in 10cm dishes and incubated with serum-fee medium for 24 hours. And then the culture medium with different doses of SAMe was used to incubate with these cells for 24 hours at 37 ℃, 5% CO_2_. We harvested the cells at 70%-80% confluence and fixed them by adding 70% ice-cold ethanol for 24 hours. Then cold phosphate-buffered saline (PBS) was used to wash the fixed cells and 1500μl propidium iodide (PI) solution was added. We used FACS Caliber (Becton-Dickinson) machine to detect the cell cycle. Results were analyzed further using the ModFit LT software. Experiments were repeated three times.

### Cell proliferation assay

HepG2 and AML12 cells were seeded in the 96 well assay plate and incubated with the medium in the presence or absence of SAMe for 48 hours. The cell viability was measured by MTS analysis using CellTiter 96 AQueous One Solution Cell Proliferation Assay (Promega). For the MTS analysis, each well of the 96-well plate was added with 100μl of cells in culture medium. After 20μl reagent was pipetted into each well, the plates were incubated for 2 hours (37 ℃, 5% CO_2_). Finally, the optical density (OD) was observed using a multi-well plate reader (AWARE-NESS) by measuring absorbance at 490nm. The OD at 490 nm was measured for detecting the cell viability. Experiments were repeated three times and operated in triplicate.

### Statistical analysis

Statistical analysis was performed with SPSS 13.0 for significance. The analysis of variance and t-test were applied in comparing the intergroup difference of experimental data. A *p*<0.05 was considered to indicate statistically significant result.

## Results

### Differentially expressed genes

In this study, we verified a transcriptome profile of the human hepatocellular carcinoma cell line HepG2 with SAMe treatment by RNA sequencing (RNA-Seq). A total of 472 genes were detected for expressed differentially (**Supplementary Table S1**), including 236 upregulated genes and 236 downregulated genes with fold changes≥2 in SAMe treated group compared with matched control group.

### Pathway and functional enrichment analysis for RNA-seq

**Figure 1:**
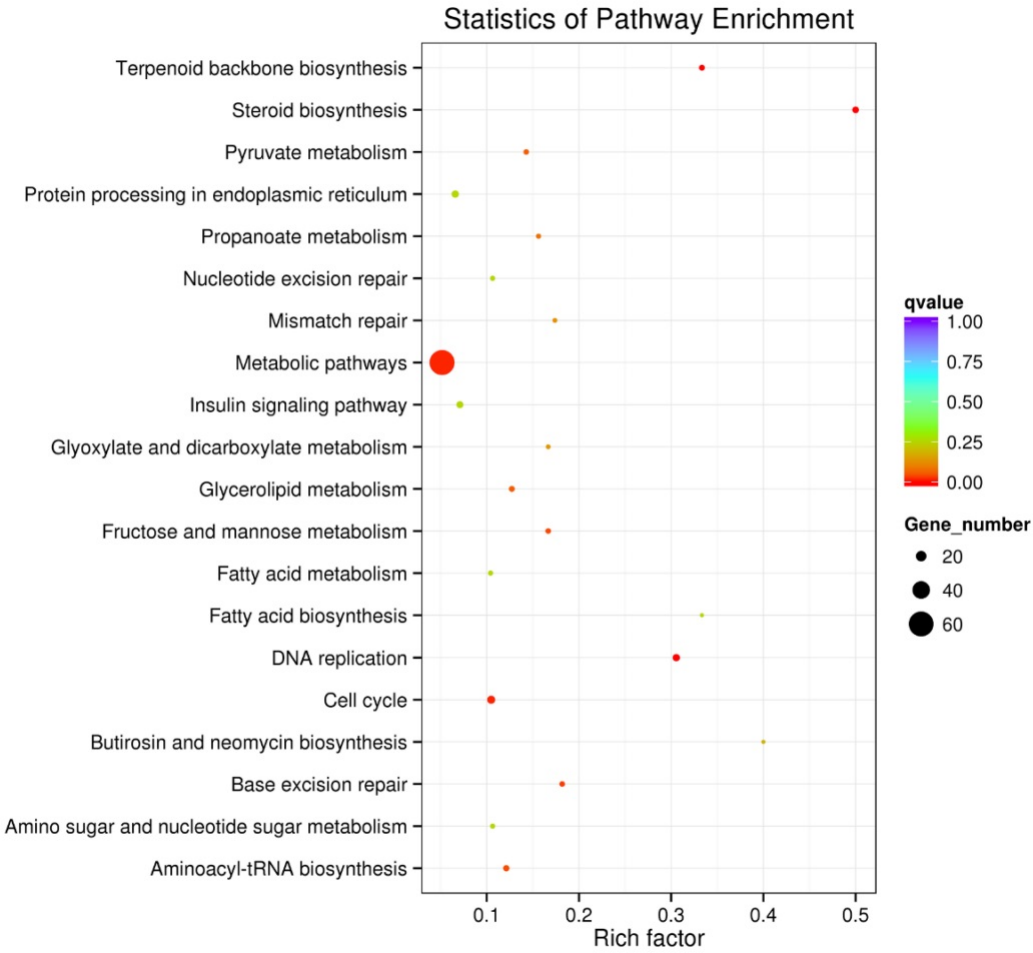
** The top 20 pathways in KEGG annotation results.** We summarized the 20 pathways with the greatest significance within this experiment. The vertical axis represents the name of pathways, and the horizontal axis represents the rich factor. The size of the dots reflects the number of the differentially expressed genes in the pathway, and the color of them represent the spectrum from week to strong scores of Qvalue.

All of the differentially expressed genes were analyzed by KEGG enrichment analysis and they were functionally assigned to 210 KEGG pathways. The majority of the significant pathways were observed in Steroid biosynthesis, DNA replication, Terpenoid backbone biosynthesis and cell cycle. The 20 pathways with the greatest significance in SAMe treatment compared to control are summarized in **Figure 1**.

**Figure 2:**
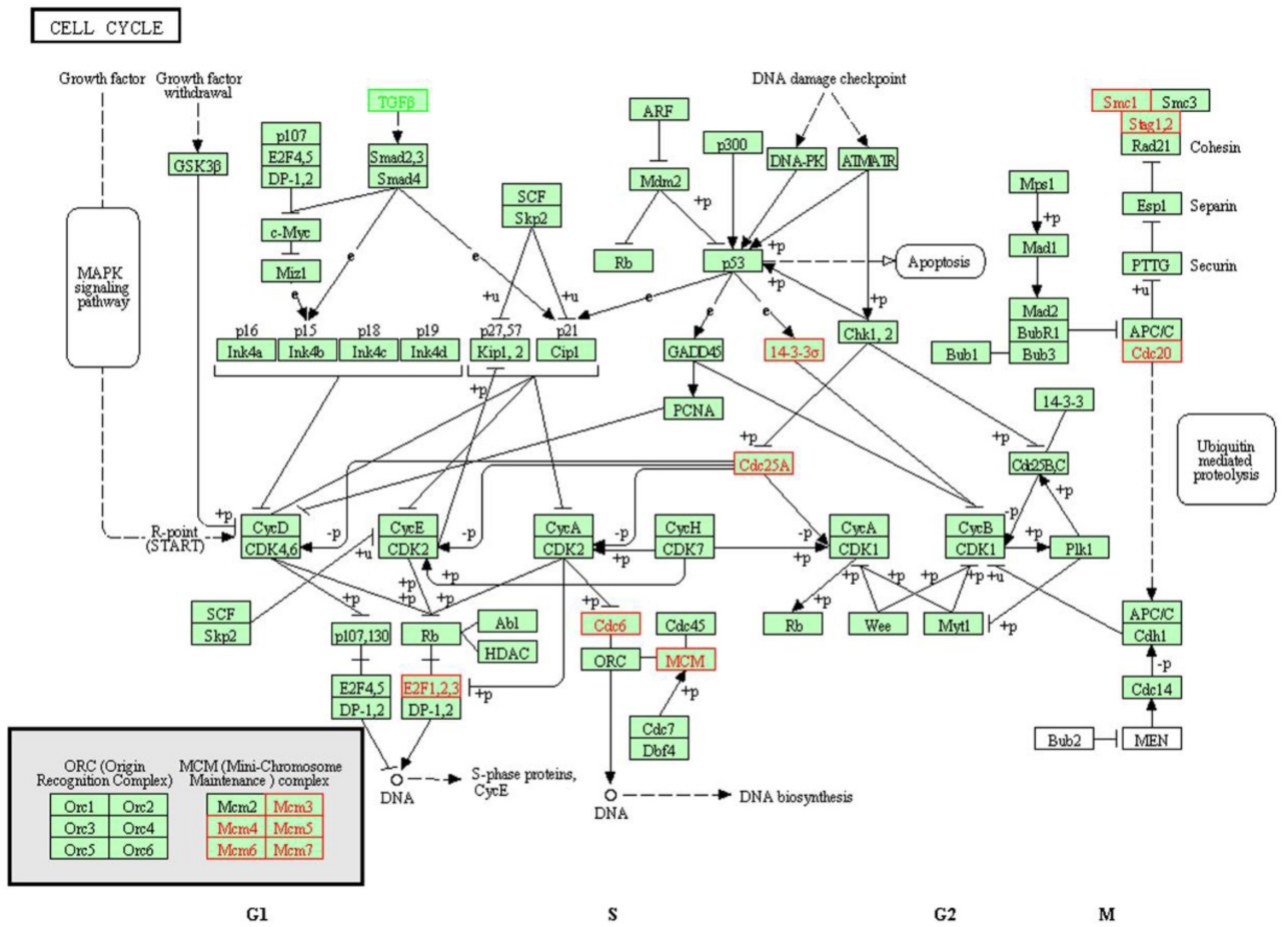
** Differentially expressed genes involved in cell cycle pathway.** There were 13 genes differentially expressed involved in the cell cycle pathway. Except TGFB1 (marked green) was downregulated, the expression of others (marked red) were accelerated.

As the dysregulation of cell cycle progression is closely associated with the tumorigenesis, and the differentially expressed genes regulated by SAMe in hepatocellular carcinoma HepG2 cells were enriched in cell cycle pathway obviously, we focused on the further study on cell cycle pathway. There were 13 genes differentially expressed involved in this pathway (**Table 1**). Except TGFB1 was downregulated, the expression of others was accelerated. Previous studies have confirmed that these genes play very important roles in the cell cycle pathway (**Figure 2**).

**Figure 3:**
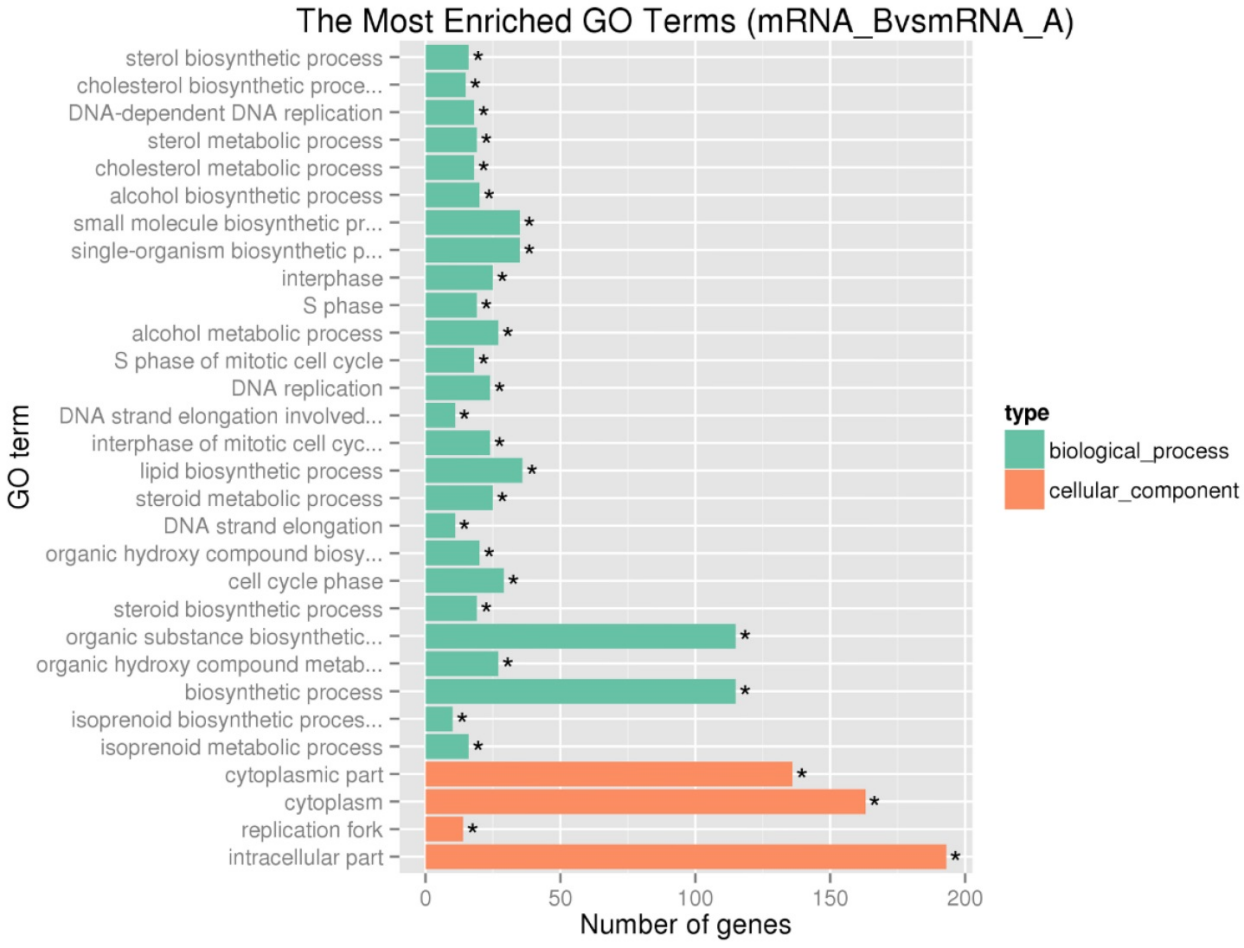
** The top 30 enriched gene ontology terms for upregulated differentially expressed genes.** We summarized the most enriched 30 GO terms in the categories for upregulated genes. The vertical axis represents the name of GO terms, and the horizontal axis represents the number of differential expressed genes within this term. *, Corrected *P-value*<0.05.

GO enrichment analysis demonstrated that 4049, 451 and 896 terms in the biological process, cellular component and molecular function categories, respectively. The most enriched GO terms in the categories for upregulated genes were included biological process related with cell cycle, such as interphase, S phase and cell cycle phase (**Figure 3**). The result was in accordance with the pathway enrichment analysis and indicated that SAMe could affect human hepatocellular carcinoma HepG2 cells by regulating the expression of genes related to cell cycle.

### Network and module analysis

**Figure 4:**
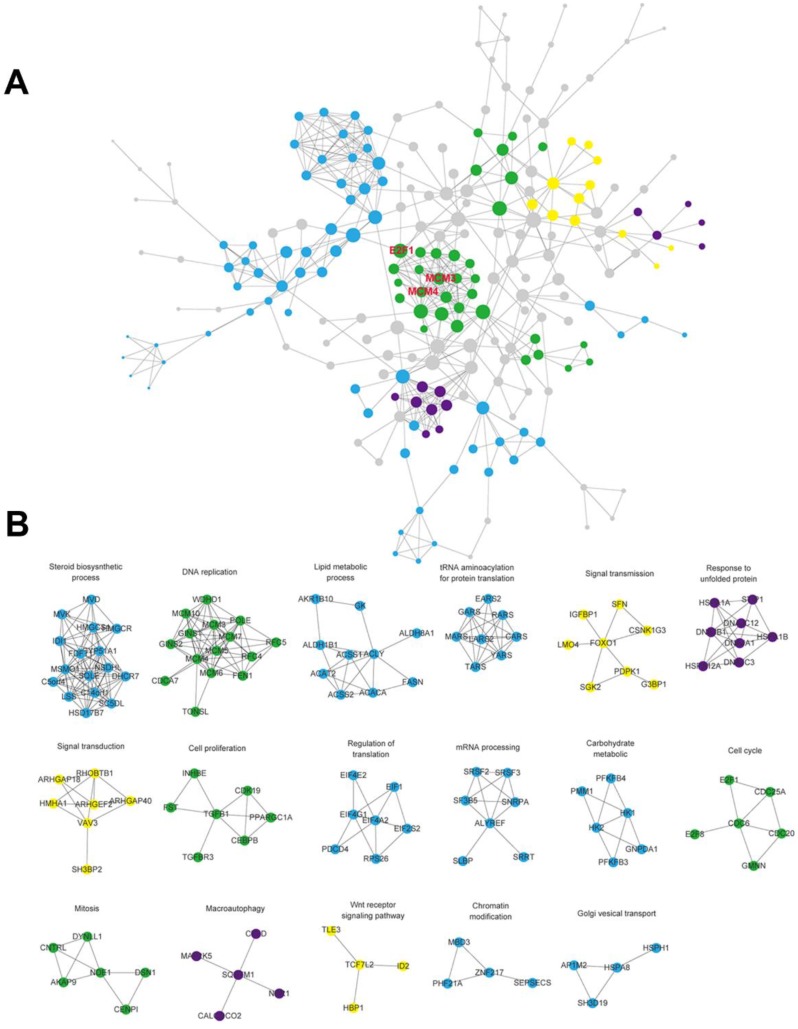
** Constructed protein-protein interaction network for the differentially expressed genes**. A. The network included interactions between all of the differential expressed genes. The size of the dots reflects the between centrality of the gene within the network. MCM3, MCM4 and E2F1 which were genes in cell cycle pathway observed within the module in the core of the network. B. Some genes with similar function were clustered into 17 modules. And the modules were divided into four categories according to their biological functions as follows: cell cycle, signal pathway, metabolism of biological macromolecules and autophagy, then marked with green, yellow, blue and purple, respectively.

The interaction network between protein and protein was visualized after the interaction of the differentially expressed genes were predicted using the STRING database (**Figure 4A**). Some genes with similar function were clustered into 17 modules. (**Figure 4B**). There existed close interactions between these modules, and they may participate in the same biological functions. These modules were divided into four categories according to their biological functions as follows: cell cycle, signal pathway, metabolism of biological macromolecules and autophagy, then marked with green, yellow, blue and purple, respectively. We found the module of cell cycle which connects different parts of the whole network is in the core of the network. It implied that cell cycle pathway may be very important to the regulation of SAMe on HepG2 cells.

### Identification of differentially expressed genes in cell cycle pathway

**Figure 5:**
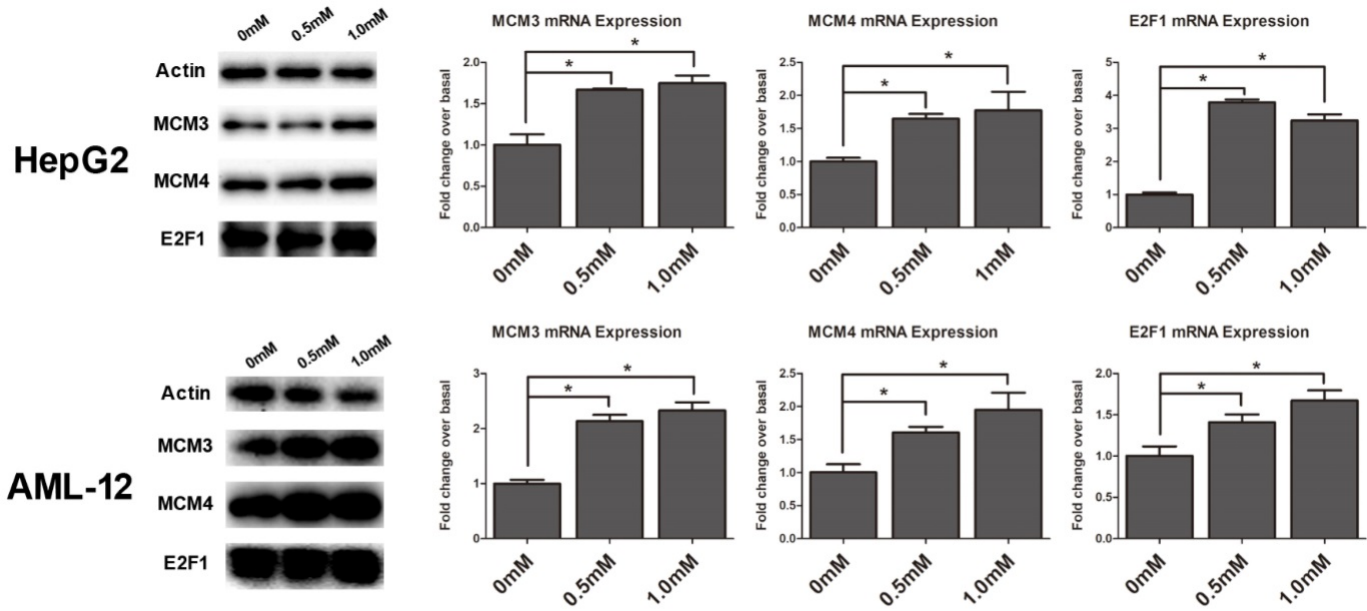
** SAMe induced up-regulation of MCM3, MCM4 and E2F1 genes and proteins.** HepG2 and AML12 cells were treated with SAMe with different does (0mM, 0.5mM, 1.0mM), and replicate cultures were harvested at 24 hours after compound addition. The total protein extracts of cells and detected by Western blot analysis with antibodies against MCM3, MCM4 and E2F1. And the total RNA was extracted for subsequent Quantitative RT-PCR. Relative protein levels and mRNA (bar graphs) were assessed. *, *p*<0.05.

To validate the gene expression profiles detected by RNA-Seq analysis, the RNA and protein expression of the genes were examined by Quantitative RT-PCR and Western blot analysis in HepG2 and AML12 cells, respectively. We found MCM3, MCM4 and E2F1 were obviously upregulated in cell cycle pathway by KEGG analysis and they were within the module in the core of the protein-protein interaction network (**Figure 4A**), so we chose them to verify. Cells were exposed to SAMe for 24 hours with different doses (0mM, 0.5mM, 1.0mM) and assessed for the genetic expression (**Figure 5**). MCM3, MCM4 and E2F1 all demonstrated significant induction by SAMe, approximately 2-fold increase in protein level was observed after SAMe treatment both in HepG2 and AML12 cells (*p*<0.05). And the expression of these proteins obviously accelerated in a dose-dependent manner. Activation of these three genes by SAMe was also probed at the mRNA level (*p*<0.05). The results confirmed the differentially expressed genes within cell cycle pathway were regulated by SAMe in liver cells.

### SAMe profoundly affects the cell division cycle in HepG2 and AML12 cells

**Figure 6:**
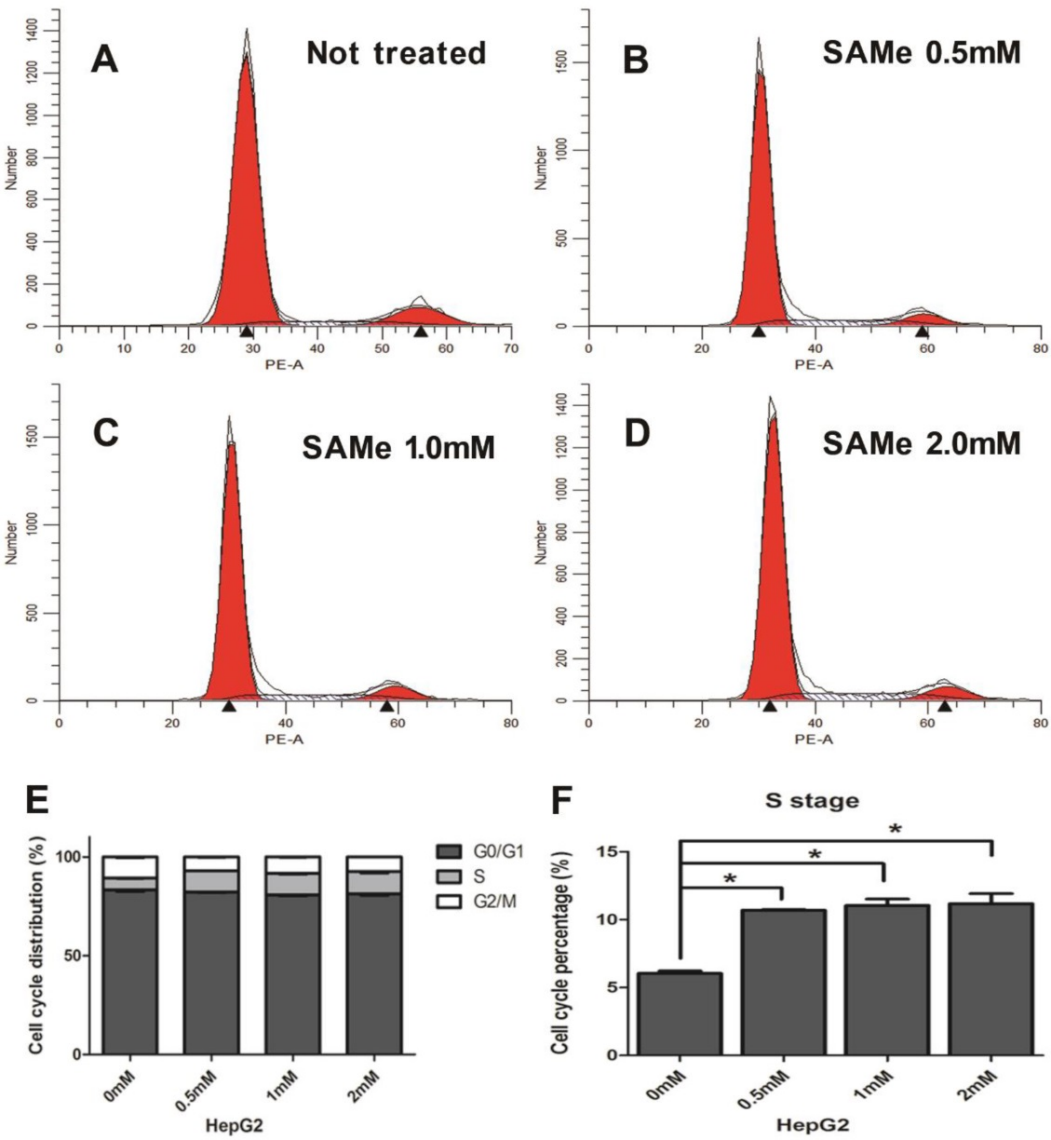
** Effect of SAMe on cell cycle in HepG2 cells.** Cells were incubated with medium supplemented or not with SAMe (A. 0mM, B. 0.5mM, C. 1.0mM and D. 2.0mM) for 24 hours. Flow cytometric analysis was performed. E&F. The percentage of S phase cells treated with SAMe is obviously more than that of control untreated cells. *, *p*<0.05.

Since SAMe could regulated the expression of genes within cell cycle pathway in liver cells, then we further explore the effects of SAMe on distribution of the cell cycle phases in HepG2 and AML12 cells by flow cytometric analysis. Cells were exposed to SAMe for 24 hours with different doses (0mM, 0.5mM, 1.0mM and 2.0mM) and assessed for the cell cycle phases. **Figure 6** shows that at different dose of SAMe (from 0.5mM up to 2.0mM), the percentage of S phase cells in SAMe incubated HepG2 is obviously more than that of control untreated cells.

**Figure 7:**
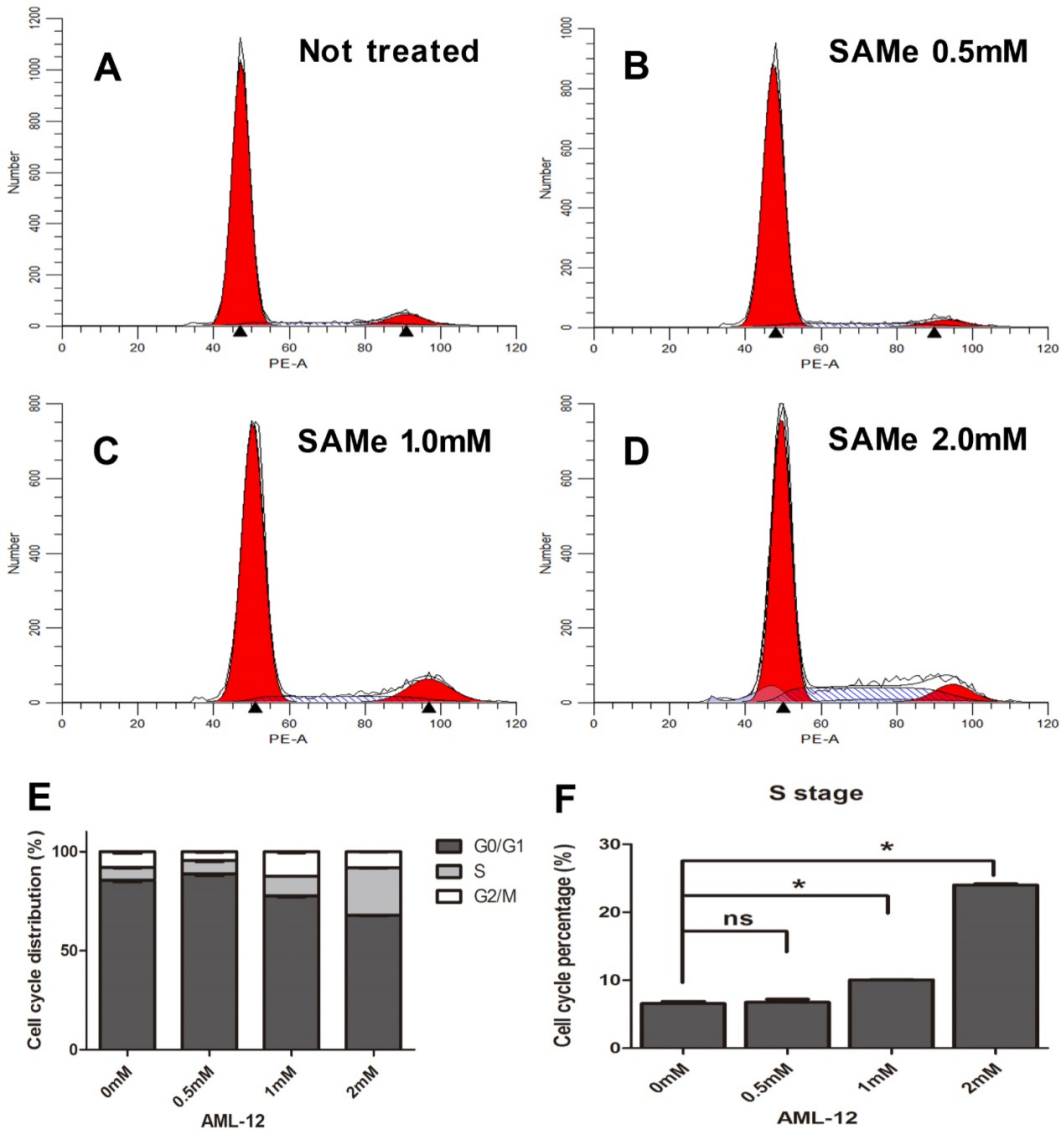
** Effect of SAMe on cell cycle in AML12 cells.** Cells were incubated with medium supplemented or not with SAMe (A. 0mM, B. 0.5mM, C. 1.0mM and D. 2.0mM) for 24 hours. Flow cytometric analysis was performed. E&F. The percentage of S phase cells treated with SAMe is obviously more than that of control untreated cells. *, *p*<0.05.

And the analysis of cell cycle distribution on SAMe treated AML12 cells also demonstrated a significant increase in S phase (from 1.0mM up to 2.0mM) compared to control group of cells (**Figure 7**). Moreover, the number of S phase cells significantly accelerated in a dose-dependent manner (**Figure 6F, 7F**). Thus, SAMe not only regulated the expression of genes, but also inhibited the progression of cell cycle through arresting cells at S phase.

### SAMe inhibits proliferation of HepG2 and AML12 cells

**Figure 8:**
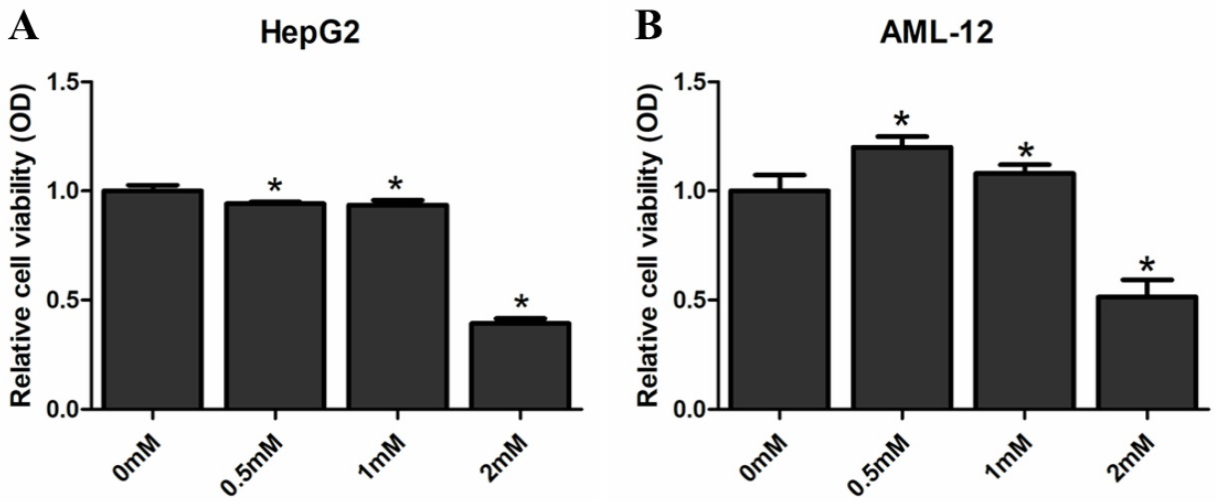
** Effect of SAMe on the proliferation of HepG2 and AML12 cells.** Cells were treated with medium supplemented with SAMe (0mM, 0.5mM, 1.0mM and 2.0mM) for 48 hours. MTS analysis was performed to measure the effect of SAMe on cell proliferation. *, *p<0.05* (vs 0 mM).

We examined the proliferative impact of SAMe on HepG2 and AML12 cells in order to evaluate the effects of SAMe on the liver cells. Cells were treated with SAMe (0 mM, 0.5 mM, 1.0 mM and 2.0 mM) for 48 hours and then MTS analysis was applied to detected the cell proliferation (**Figure 8**). SAMe treatment caused a dose-dependent inhibition of HepG2 cell proliferation; SAMe treatment did not inhibit AML12 proliferation at the concentration of 0.5 mM and 1.0 mM, while inhibited AML12 proliferation at the concentration of 2.0 mM. At the concentration of 2mM, the cell viability of AML12 (0.514±0.079) was higher than HepG2 (0.393±0.023). Taken together, our results suggested that SAMe treatment could inhibit the cell proliferation of HepG2 and AML12 cells by slowing-down cell cycle, and this effect is more obvious in HepG2 than AML12 cells.

## Discussion

Hepatocellular carcinoma (HCC) is one of the most common gastrointestinal malignancies worldwide. Patients with advanced HCC possess a dismal poor prognosis with their median survival times are generally less than one year 5. Even the patients could undergo surgery, the 2 year recurrence rate still up to 50% 32. Conventional chemotherapy is not only proved to be ineffective for HCC, but also exists serious toxicity, and it is rarely used for treatment 33. Therefore, novel therapeutic approaches and agents to HCC are urgently needed.

S-Adenosylmethionine (SAMe) is well known as the principal biological methyl donor. It is importance for regulating multiple hepatic functions 34 and SAMe synthesis is reduced in chronic liver disease 14. SAMe is also available as a drug in many parts of the world in the treatment of various forms of chronic liver dysfunction such as alcoholic liver injury 35, intrahepatic cholestasis 36, and so on. SAMe at pharmacological doses has no toxic effects toward normal liver cells 37,23. Recent researches illustrate that SAMe plays an essential role in diverse cellular processes including cell growth and death, even contribute to hepatocarcinogenesis. One important molecular mechanisms about growth inhibitory effect is SAMe can suppress the mitogenic activity of growth factors 38, 39. Ansorena E 37 have reported that SAMe could induced apoptosis in HCC cells, while it protected against okadaic acid-induced apoptosis in normal hepatocytes. Lu SC and her colleagues proved that SAMe was capable of inhibiting the establishment of HCC model and exhibited anti-angiogenic properties 24. All of these evidences indicate that SAMe might be effective in preventing HCC. Nevertheless, the efficacy and the mechanisms behind it are not clearly elucidated at present.

Increased SAMe levels could induce genomics alterations in human hepatoma cells. In the current study, we used RNA-Seq to identified 472 differentially expressed genes in SAMe treated HepG2 cells compared to the control untreated cells, including 236 upregulated genes and 236 downregulated genes. To make further understanding of the transcriptome data, KEGG pathway and GO enrichment analysis were applied. The differentially expressed genes were functionally assigned to 210 KEGG pathways, including Steroid biosynthesis, DNA replication, Terpenoid backbone biosynthesis and cell cycle with the greatest significance. And the result of GO enrichment analysis was in accordance with the pathway analysis and demonstrated the most enriched terms in the categories for upregulated genes were included biological process related with cell cycle. Through the construction of protein-protein interaction network, we observed the module associated with cell cycle is in the middle of the whole network. All these results implied that cell cycle pathway may be very important to the regulation of SAMe effected on HepG2 cells.

A normal cell cycle is controlled by numerous mechanisms. And some dysregulations within cell cycle could lead to aberrant cell proliferation and develop to cancer eventually 40. Considered the differentially expressed genes regulated by SAMe in HepG2 cells were enriched in cell cycle pathway obviously, we inferred adjusting to cell cycle is essential for the potential inhibitory effect of SAMe in hepatoma cells. There were 13 genes differentially expressed involved, and most of them were accelerated except TGFB1. These significant genes have been proved to play very important roles in cell cycle regulation. MCM3, MCM4 and E2F1 were obviously upregulated in cell cycle pathway and they were found in the middle module of the protein-protein interaction network, so we chose them to validate the gene expression profiles detected by RNA-Seq analysis. We found that MCM3, MCM4 and E2F1 all demonstrated significant induction by SAMe at both mRNA and protein levels (*p*<0.05), which were in accordance with the RNA-Seq result. And the expression of these three genes obviously accelerated in a dose-dependent manner. The results confirmed the differentially expressed genes within cell cycle pathway were regulated by SAMe in liver cells.

Minichromosome maintenance family (MCM) which contains six highly related MCM genes (MCM2-7) is very important to DNA replication 41. Many researches have reported that MCM genes play essential roles in various tumors 42,43. But comprehensive analyses of the diagnostic and prognostic values of MCM genes in HCC still not clear. Xiwen Liao^44^ observed that the expression of MCM2-7 genes was increased in HCC tissue, but only MCM2, MCM6 and MCM7 were significantly correlated with the HCC overall survival. Nan YL^45^ reported the HCC risk was lower in patients with the MCM4. The E2F1 transcription factor has been identified as a tumor-suppressor gene enhancing apoptosis by DNA damage in tumors 46. Some researchers found that downregulation of E2F1 may be a key factor in the inhibitory effects in HCC 47,48. In our study, we found MCM3, MCM4 and E2F1 all demonstrated significant induction by SAMe, and inferred that they might be involved in the antiproliferative effect in response to SAMe.

To explore further explain the roles of SAMe on the cell cycle in liver cells, we examined the distribution of the cell cycle phases and the proliferative impact responded to SAMe treatment in HepG2 and AML12 cells. Our study exhibits that SAMe treatment possessed a significant effect on reducing liver cells proliferation and inhibiting the progression of cell cycle through arresting cells at S phase. The number of S phase cells significantly accelerated in a dose-dependent manner. And these effects were more obvious in HepG2 cells than AML-12 cells. Previous investigation found treatment of human colorectal cells SW-620 with SAMe could also decreased cell proliferation but altered cell cycle arrest at G2/M phase 18. Surabhi Parashar 49 reported that SAMe is effective in inhibiting tumor growth of human osteosarcoma cells and increasing the number of tumor cells in G2/M phase. These results are differed from ours because the effect of SAMe on cell cycle regulation might be cell-type specific. But accumulating evidence shows that SAMe regulates proliferation in various tumors.

Although more studies are required to further understand the involvement of SAMe treatment in HCC, we provide evidence that SAMe plays an important role of antiproliferative action in liver cells, capable of regulating genes involved in cell cycle pathway leading to proliferation inhibition, and can serve as a possible anticancer agent used in hepatocellular carcinoma therapy.

## Supplementary Material

Supplementary table S1.

## Figures and Tables

**Table A:** Primers

Gene name	Forward	Reverse
MCM3 (human)	CCTTGGGTAGTGCTGTGGAT	AGTGTTCGGGCTGTAACTGG
MCM4 (human)	AGCATGGCACTCATCCACAA	GCACAGCTCGATAGATGCCT
E2F1 (human)	AAACAAGGCCCGATCGATGT	TGGGATCTGTGGTGAGGGAT
β-Acting (human)	AGGGGCCGGACTCGTCATACT	GGCGGCACCACCATGTACCCT
MCM3 (mouse)	CCACCTACGCCAAGCAGTAT	CCTTCCACACAGACCACACA
MCM4 (mouse)	CTTGTTTTCCAGCCCTCCTC	CCACTTCTTGGGGTTCCTTC
E2F1 (mouse)	ATCACCTCCCTCCACATCC	TGACAGTTGGTCCTCTTCCA
β-Acting (mouse)	GGCCAACCGTGAAAAGATGA	CACAGCCTGGATGGCTACGTA

**Table 1:** Differnetial expressed genes associated with cell cycle.

Gene symbol	Gene title	log2 Fold Change	*p* value	Adjusted* p*
CDC6	Cell division cycle 6	1.6653	4.26E-15	5.77E-12
SFN	Stratifin	1.587	1.69E-08	0.00000776
E2F1	E2F transcription factor 1	1.1015	2.14E-07	0.0000693
CDC25A	Cell division cycle 25A	1.4223	0.000001	0.00024731
MCM6	Minichromosome maintenance complex component 6	1.384	1.59E-06	0.00034698
MCM4	Minichromosome maintenance complex component 4	1.3216	1.64E-06	0.00035229
STAG1	Stromal antigen 1	1.1586	4.82E-06	0.00086294
TGFB1	Transforming growth factor beta 1	-0.76717	0.0001565	0.014454
MCM3	Minichromosome maintenance complex component 3	1.1317	0.000205	0.017534
MCM5	Minichromosome maintenance complex component 5	1.017	0.0004547	0.03076
MCM7	Minichromosome maintenance complex component 7	0.91777	0.0007119	0.04129
CDC20	Cell division cycle 20	0.69303	0.0007335	0.042275
SMC1A	Structural maintenance of chromosomes 1A	0.65421	0.0007941	0.044867
