# Supplementary material for: S-Adenosylmethionine Affects Cell Cycle Pathways and Suppresses Proliferation in Liver Cells
Source: J Cancer. 2019 Jul 22;10(18):4368–79. doi: 10.7150/jca.25422 (PMC6691693; doi:10.7150/jca.25422)
Supplement: Supplementary file 1 — Supplementary table S1. [file jcav10p4368s1.pdf]

	Gene_id	readcount_mRNA_B	readcount_mRNA_A	log2FoldChange	Fold	pval	padj
1	ENSG00000001461	41.57392247	106.8615289	-1.362	0.3890426	2.88E-05	0.00367
2	ENSG00000001630	168.3328935	78.84030233	1.0943	2.1350946	5.54E-05	0.00629
3	ENSG00000003989	723.7771517	2042.43168	-1.4967	0.354363	1.04E-05	0.00166
4	ENSG00000004866	89.59859278	36.42700067	1.2985	2.4597301	0.000163	0.01479
5	ENSG00000006459	231.2354903	566.5018696	-1.2927	0.4081864	0.0003923	0.02769
6	ENSG00000011021	529.2656661	292.6092431	0.85502	1.8087838	2.28E-05	0.00307
7	ENSG00000011258	127.7127485	296.7909253	-1.2165	0.4303254	2.87E-07	8.81E-05
8	ENSG00000011376	352.4934394	209.9059743	0.74785	1.6792884	0.0007805	0.04439
9	ENSG00000023287	281.7418408	492.4563673	-0.80562	0.5721162	0.0001214	0.01203
10	ENSG00000033100	397.6081591	183.939622	1.1121	2.1616006	3.48E-07	0.00011
11	ENSG00000034063	327.3091401	93.13540132	1.8133	3.5144526	3.27E-09	1.83E-06
12	ENSG00000039523	154.4857234	297.6438783	-0.94611	0.5190301	5.27E-05	0.00606
13	ENSG00000044574	8355.481852	5492.982039	0.60513	1.5211158	0.0006328	0.0373
14	ENSG00000046604	645.1968088	1053.086279	-0.70681	0.6126733	0.0002233	0.01856
15	ENSG00000047346	20.28981489	56.40010508	-1.4749	0.3597583	0.0004284	0.02967
16	ENSG00000051180	180.2765602	96.45023692	0.90236	1.869121	0.0006008	0.03625
17	ENSG00000052802	1567.969057	501.2058996	1.6454	3.1283458	8.07E-18	1.60E-14
18	ENSG00000053372	449.2141419	261.8334687	0.77875	1.7156437	0.0008427	0.0471
19	ENSG00000056736	1130.604206	563.8123087	1.0038	2.0052749	2.52E-07	7.89E-05
20	ENSG00000058262	3105.294978	1903.442649	0.70612	1.6314107	6.62E-05	0.00739
21	ENSG00000059145	254.1285311	121.8034715	1.061	2.0863772	0.0002587	0.02077
22	ENSG00000060982	412.9872835	678.7150129	-0.71671	0.6084835	0.0003195	0.02386
23	ENSG00000062822	424.813456	256.5503147	0.72759	1.6558707	0.0009038	0.04954
24	ENSG00000065060	375.7742354	692.0549726	-0.88102	0.5429834	8.93E-06	0.00148
25	ENSG00000065328	281.256849	109.4325447	1.3618	2.5700564	2.83E-08	1.19E-05
26	ENSG00000065911	479.7171446	1748.685502	-1.866	0.274333	2.20E-20	5.15E-17
27	ENSG00000066027	109.419622	200.8957661	-0.87658	0.544657	0.0005652	0.03476
28	ENSG00000067064	1261.85084	263.7420105	2.2583	4.7842739	1.46E-28	6.29E-25
29	ENSG00000068650	836.8429105	1319.688497	-0.65717	0.634121	0.0004913	0.03204
30	ENSG00000068654	1093.165464	693.776509	0.65597	1.575675	0.0008122	0.04579
31	ENSG00000069702	885.1702541	1864.254154	-1.0746	0.4748027	1.05E-08	5.09E-06
32	ENSG00000071539	311.5544151	176.0976092	0.82311	1.7692158	0.0005225	0.03283
33	ENSG00000071655	392.6529018	218.9161825	0.84288	1.7936271	0.0001617	0.01479
34	ENSG00000072422	514.8038555	266.3589093	0.95065	1.9327433	7.42E-06	0.00127
35	ENSG00000072501	1076.860922	684.2640529	0.65421	1.573754	0.0007941	0.04487
36	ENSG00000072864	276.8068411	562.3782273	-1.0227	0.4921943	0.0008301	0.04649
37	ENSG00000075336	235.204558	122.4642089	0.94156	1.9206039	0.000162	0.01479
38	ENSG00000075420	1285.554664	2265.344276	-0.81734	0.5674873	9.78E-06	0.00158
39	ENSG00000075702	251.4969445	130.7149669	0.94412	1.9240149	0.0001125	0.01141
40	ENSG00000076003	498.2331049	190.894956	1.384	2.6099099	1.59E-06	0.00035
41	ENSG00000076248	745.1665989	303.4007644	1.2963	2.455982	2.50E-06	0.0005
42	ENSG00000076356	99.48479825	252.6717178	-1.3447	0.3937359	4.57E-08	1.81E-05
43	ENSG00000077312	240.1476607	412.2626009	-0.77964	0.5825121	0.0002543	0.02054
44	ENSG00000079385	455.4825712	760.1176474	-0.73883	0.5992251	0.000187	0.01639
45	ENSG00000079432	589.0395301	355.1610856	0.72989	1.6585126	0.0003963	0.0279
46	ENSG00000079459	4449.831789	1783.531361	1.319	2.4949311	3.84E-13	4.13E-10
47	ENSG00000083799	67.29993491	134.9390587	-1.0036	0.4987539	0.0004166	0.02893
48	ENSG00000084731	106.9663025	241.99975	-1.1778	0.442025	2.21E-06	0.00045
49	ENSG00000086061	2079.858911	755.7256541	1.4606	2.752228	3.89E-11	3.23E-08
50	ENSG00000087087	1275.75354	550.6534159	1.2121	2.3167462	0.000227	0.01879
51	ENSG00000087266	143.56471	519.7622898	-1.8562	0.2762028	1.27E-05	0.00194
52	ENSG00000088826	250.6687643	417.0452439	-0.73442	0.6010596	0.0004767	0.03171
53	ENSG00000088854	217.3721141	377.490413	-0.79627	0.575836	0.0003029	0.02302
54	ENSG00000088986	1631.2351	578.3559266	1.4959	2.8204004	9.63E-15	1.24E-11
55	ENSG00000090339	493.6656028	1113.948575	-1.1741	0.4431601	6.27E-08	2.37E-05
56	ENSG00000091157	87.65052266	38.64036451	1.1817	2.2684392	0.0005253	0.03293
57	ENSG00000094804	706.664688	222.791306	1.6653	3.171796	4.26E-15	5.77E-12
58	ENSG00000095380	507.1574299	296.6991594	0.77343	1.7093289	0.0002707	0.02133
59	ENSG00000095574	61.40710626	135.236934	-1.139	0.4540742	0.0001104	0.0113
60	ENSG00000096696	5729.61803	8956.951407	-0.64457	0.6396834	0.0003336	0.02477
61	ENSG00000100292	249.6785232	112.3507922	1.1521	2.2223715	0.0003591	0.02613
62	ENSG00000100297	652.1528061	322.2671869	1.017	2.0237064	0.0004547	0.03076
63	ENSG00000100307	18.7862213	84.06888979	-2.1619	0.2234618	0.0004753	0.03171
64	ENSG00000100344	305.4549587	142.3827467	1.1012	2.1453306	1.76E-06	0.00038
65	ENSG00000100395	231.2192842	108.5284986	1.0912	2.1305117	1.88E-05	0.00261
66	ENSG00000100417	109.9127168	222.1781883	-1.0154	0.4946912	4.86E-05	0.00564
67	ENSG00000100462	1014.55676	619.4070806	0.71189	1.6379485	0.0002604	0.02077
68	ENSG00000101003	460.6079925	220.8775541	1.0603	2.0853651	1.39E-06	0.00032
69	ENSG00000101049	145.0926128	61.53171643	1.2376	2.3580593	1.42E-05	0.00209

70	ENSG00000101363	397.0097247	241.3494329	0.71805	1.6449571	0.0008559	0.04755
71	ENSG00000101412	807.5842039	376.3517418	1.1015	2.1457768	2.14E-07	6.93E-05
72	ENSG00000101670	49.49990324	7.295618762	2.7623	6.7847704	1.56E-07	5.22E-05
73	ENSG00000102384	125.7727814	50.01374255	1.3304	2.5147239	3.89E-05	0.00466
74	ENSG00000102572	608.5376284	1011.009789	-0.73238	0.6019101	0.0004775	0.03171
75	ENSG00000102580	752.6521546	380.6095596	0.98367	1.9774895	6.39E-07	0.00017
76	ENSG00000102753	550.1417609	333.7305941	0.72112	1.6484613	0.0005989	0.03622
77	ENSG00000102934	292.0806259	506.8553893	-0.79521	0.5762593	0.0001014	0.01054
78	ENSG00000103356	496.5512443	258.3566701	0.94258	1.9219623	8.40E-06	0.0014
79	ENSG00000104549	2728.049561	969.8227226	1.4921	2.8129814	8.54E-16	1.38E-12
80	ENSG00000104738	1916.589474	766.8046304	1.3216	2.4994315	1.64E-06	0.00035
81	ENSG00000104765	376.2997424	615.7683189	-0.71051	0.6111041	0.0004878	0.03204
82	ENSG00000104976	71.29736325	142.4355766	-0.99839	0.5005583	0.0003523	0.02579
83	ENSG00000105227	8.904067359	38.74081407	-2.1213	0.2298397	7.38E-05	0.00806
84	ENSG00000105281	1609.70385	2954.520946	-0.87613	0.544827	1.12E-06	0.00027
85	ENSG00000105329	354.0942696	602.6414153	-0.76717	0.5875689	0.0001565	0.01445
86	ENSG00000105856	68.82378612	172.1150893	-1.3224	0.3998692	3.68E-06	0.0007
87	ENSG00000105939	509.5823887	247.5929363	1.0413	2.0580813	1.15E-06	0.00027
88	ENSG00000106105	769.5348726	1806.444282	-1.2311	0.4259925	7.26E-11	5.84E-08
89	ENSG00000106351	776.7206156	1316.308675	-0.76103	0.5900749	6.48E-05	0.00726
90	ENSG00000108175	533.2549914	847.8555514	-0.66899	0.6289468	0.0006851	0.04002
91	ENSG00000108176	3.965016148	23.89758428	-2.5915	0.1659131	0.0003869	0.02746
92	ENSG00000108179	1646.036048	893.1637949	0.882	1.8429284	3.18E-06	0.00062
93	ENSG00000108244	484.4142955	855.6516812	-0.82078	0.5661358	3.36E-05	0.00417
94	ENSG00000108312	442.6337453	212.4752534	1.0588	2.083198	1.49E-06	0.00033
95	ENSG00000108405	0.49714634	17.50774831	-5.1382	0.0283954	0.0003787	0.02717
96	ENSG00000108592	767.2748351	437.3283806	0.81103	1.7544636	6.38E-05	0.00717
97	ENSG00000108826	380.2363979	216.9530743	0.80951	1.7526161	0.0002938	0.02246
98	ENSG00000109066	295.6416807	105.5080648	1.4865	2.8020836	1.24E-09	7.82E-07
99	ENSG00000109084	1260.120361	559.2903417	1.1719	2.2530823	2.04E-09	1.20E-06
100	ENSG00000109618	72.7766477	151.8917293	-1.0615	0.4791336	0.0001475	0.01392
101	ENSG00000109686	421.8913508	924.8756045	-1.1324	0.4561563	3.12E-06	0.00062
102	ENSG00000109819	75.22996721	337.0808924	-2.1637	0.2231831	9.89E-05	0.0104
103	ENSG00000109929	966.1889014	397.1162857	1.2827	2.4329387	7.63E-11	5.95E-08
104	ENSG00000109971	11175.74253	3296.781349	1.7612	3.3897996	8.94E-22	2.56E-18
105	ENSG00000110172	572.0161999	234.6088918	1.2858	2.4381722	1.14E-09	7.35E-07
106	ENSG00000110245	357.8972248	205.7823321	0.79843	1.7392074	0.0003808	0.02725
107	ENSG00000110619	178.2717688	500.3035903	-1.4887	0.3563335	8.83E-12	8.42E-09
108	ENSG00000110721	979.8578044	609.9509279	0.68388	1.6064544	0.0005011	0.03225
109	ENSG00000110921	537.6725872	108.6783046	2.3067	4.947501	1.38E-23	4.45E-20
110	ENSG00000111077	158.4345335	310.9768912	-0.97292	0.5094738	2.73E-05	0.00352
111	ENSG00000111252	106.4691561	271.435954	-1.3502	0.3922377	2.80E-08	1.19E-05
112	ENSG00000111371	853.9472712	1754.294319	-1.0387	0.4867659	2.51E-08	1.10E-05
113	ENSG00000111412	442.6175392	224.3032596	0.98061	1.9732996	0.0002416	0.01963
114	ENSG00000111445	225.2818888	107.8747081	1.0624	2.0884028	3.52E-05	0.00433
115	ENSG00000111897	591.2631038	1017.727024	-0.78348	0.5809637	3.89E-05	0.00466
116	ENSG00000111912	147.9782542	290.9016005	-0.97515	0.508687	3.62E-05	0.00442
117	ENSG00000111961	121.7956107	222.2275447	-0.86758	0.5480654	0.0004727	0.03171
118	ENSG00000112081	2033.054227	1109.839835	0.8733	1.8318483	0.0008497	0.04738
119	ENSG00000112118	3346.506998	1527.267044	1.1317	2.1911678	0.000205	0.01753
120	ENSG00000112312	621.4512787	306.1094291	1.0216	2.0301692	1.01E-06	0.00025
121	ENSG00000112414	1013.404458	2228.260011	-1.1367	0.4547987	1.07E-09	7.05E-07
122	ENSG00000112972	3171.484317	679.4094762	2.2228	4.6679852	7.58E-32	4.88E-28
123	ENSG00000113161	2073.968942	748.6949129	1.4699	2.7700269	3.95E-15	5.65E-12
124	ENSG00000113407	584.7880468	1028.443138	-0.81448	0.5686134	1.94E-05	0.00264
125	ENSG00000113552	384.7593332	166.7895257	1.2059	2.3068113	1.03E-07	3.70E-05
126	ENSG00000113643	717.2060492	424.5382883	0.75649	1.6893755	0.000146	0.01383
127	ENSG00000113721	82.69526535	218.0597562	-1.3988	0.3792445	4.51E-06	0.00083
128	ENSG00000113739	726.3520169	1444.935041	-0.99226	0.5026897	1.12E-07	3.95E-05
129	ENSG00000114120	307.4475956	537.3774346	-0.8056	0.5721241	0.0001267	0.01251
130	ENSG00000114268	473.9782739	258.4605931	0.87488	1.8338555	4.72E-05	0.00551
131	ENSG00000114439	170.2769121	392.6419383	-1.2053	0.4336792	5.02E-08	1.96E-05
132	ENSG00000114867	4032.584919	2445.168211	0.72177	1.6492042	7.06E-05	0.00781
133	ENSG00000115053	9927.622553	6441.603966	0.62403	1.5411743	0.000514	0.03256
134	ENSG00000115107	407.8618622	241.8057978	0.75423	1.6867311	0.000547	0.03389
135	ENSG00000115306	5419.450905	9783.017078	-0.85213	0.5539663	2.01E-06	0.00042
136	ENSG00000115594	261.298068	555.8507424	-1.089	0.4700871	1.86E-07	6.15E-05
137	ENSG00000115687	343.7190208	160.1476975	1.1018	2.146223	1.64E-06	0.00035
138	ENSG00000115738	1188.681194	720.9698249	0.72135	1.6487241	0.0001111	0.01131
139	ENSG00000115758	2405.80626	1373.256182	0.80892	1.7518995	1.37E-05	0.00203

140	ENSG00000116062	817.4582548	483.9164176	0.75639	1.6892584	0.0001597	0.01469
141	ENSG00000116237	859.432087	479.0931019	0.84308	1.7938758	0.0002843	0.0222
142	ENSG00000116584	138.0839457	605.7942879	-2.1333	0.2279359	1.33E-06	0.00031
143	ENSG00000116649	717.1857916	404.4101677	0.82653	1.7734148	3.07E-05	0.00388
144	ENSG00000116761	36.62271669	118.585612	-1.6951	0.3088333	1.50E-07	5.07E-05
145	ENSG00000116830	302.5450081	176.8977323	0.77423	1.710277	0.00061	0.03663
146	ENSG00000116852	4.959308828	26.46338995	-2.4158	0.1874009	0.0003705	0.02674
147	ENSG00000117139	341.0550221	548.4512006	-0.68536	0.6218506	0.0009111	0.04973
148	ENSG00000117399	584.3435703	361.4487353	0.69303	1.6166754	0.0007335	0.04228
149	ENSG00000118007	231.2679025	103.5960497	1.1586	2.2324069	4.82E-06	0.00086
150	ENSG00000118503	412.0416091	736.8106035	-0.8385	0.5592247	0.0006609	0.03878
151	ENSG00000118514	68.81568307	8.301851118	3.0512	8.2890111	9.98E-11	7.35E-08
152	ENSG00000118515	114.928747	240.9987278	-1.0683	0.4768806	1.08E-05	0.00168
153	ENSG00000119397	120.2798625	219.2582041	-0.86624	0.5485747	0.0005348	0.03336
154	ENSG00000119737	31.20677675	7.295618762	2.0968	4.2775953	0.0004814	0.03185
155	ENSG00000119777	1374.503343	897.2985858	0.61525	1.5318234	0.0006285	0.03714
156	ENSG00000119801	81.15925957	272.4352395	-1.7471	0.2979	7.24E-07	0.00018
157	ENSG00000119812	430.8359334	185.0973971	1.2189	2.3276917	2.08E-08	9.39E-06
158	ENSG00000119950	63.88473491	238.8260368	-1.9024	0.267498	1.04E-05	0.00166
159	ENSG00000120437	1793.20924	705.8721379	1.3451	2.5404781	5.54E-07	0.00016
160	ENSG00000120694	1115.185757	337.0541133	1.7262	3.3085521	1.18E-17	2.18E-14
161	ENSG00000120738	62.87423613	236.1069517	-1.9089	0.2662955	0.0007843	0.04451
162	ENSG00000120800	558.8918704	319.2398062	0.80793	1.7506977	0.0001157	0.0116
163	ENSG00000121741	189.5359706	321.5942924	-0.76277	0.5893637	0.0007659	0.04375
164	ENSG00000122741	110.8908034	206.8803304	-0.89966	0.536013	0.0004371	0.02998
165	ENSG00000123983	1821.154774	2952.037214	-0.69686	0.6169134	0.0001504	0.01409
166	ENSG00000124143	6.438593277	29.28118789	-2.1852	0.2198818	0.0004573	0.03076
167	ENSG00000124593	81.19167176	159.5397899	-0.97451	0.5089127	0.0003495	0.02565
168	ENSG00000124788	201.9322169	415.5263434	-1.0411	0.4859568	2.14E-06	0.00044
169	ENSG00000125089	141.0789783	266.7572341	-0.91902	0.5288682	9.53E-05	0.01006
170	ENSG00000125144	115.4137387	21.58550759	2.4187	5.34689	3.78E-11	3.23E-08
171	ENSG00000125454	120.7729573	57.81160913	1.0629	2.0891267	0.000561	0.03466
172	ENSG00000125753	436.7166075	819.3831701	-0.90784	0.5329825	4.52E-06	0.00083
173	ENSG00000125772	72.80095684	182.5315914	-1.3261	0.398845	1.02E-06	0.00025
174	ENSG00000125977	930.0228158	1457.144005	-0.64781	0.6382484	0.0005268	0.03294
175	ENSG00000126391	215.9657571	121.0544416	0.83515	1.7840425	0.0009093	0.04973
176	ENSG00000126562	81.17546567	188.8192411	-1.2179	0.429908	5.35E-06	0.00094
177	ENSG00000127561	13.86742771	44.22313053	-1.6731	0.3135788	0.000622	0.03692
178	ENSG00000127663	251.5658204	425.1427224	-0.75701	0.5917214	0.000348	0.02565
179	ENSG00000127914	142.1056832	335.9811574	-1.2414	0.422962	9.38E-08	3.40E-05
180	ENSG00000128165	25.75032159	167.5420291	-2.7019	0.1536905	2.11E-17	3.63E-14
181	ENSG00000128228	343.7230723	132.2251838	1.3782	2.5994385	4.83E-09	2.49E-06
182	ENSG00000128272	939.4593021	2438.690811	-1.3762	0.3852321	1.24E-13	1.39E-10
183	ENSG00000128512	133.1813582	263.4830713	-0.98432	0.5054639	4.22E-05	0.00498
184	ENSG00000128578	73.78309495	29.1313819	1.3407	2.5327418	0.0003107	0.02344
185	ENSG00000128645	61.39090016	226.1961708	-1.8815	0.2714014	0.0005177	0.03261
186	ENSG00000129173	101.538208	46.33951824	1.1317	2.1911678	0.0005473	0.03389
187	ENSG00000129255	372.3833445	207.7454403	0.84197	1.7924961	0.0001768	0.0156
188	ENSG00000129354	365.5326869	105.3089024	1.7954	3.471117	5.31E-10	3.60E-07
189	ENSG00000129484	453.7330257	279.2849138	0.70011	1.6246287	0.0008866	0.04881
190	ENSG00000130054	119.3666003	55.1929736	1.1128	2.1626497	0.0002288	0.01883
191	ENSG00000130165	185.6762941	100.626709	0.88378	1.8452036	0.0007259	0.04193
192	ENSG00000130517	466.298245	793.2704853	-0.76656	0.5878174	0.0001076	0.01113
193	ENSG00000130589	158.3697091	282.8058586	-0.83652	0.5599927	0.0003111	0.02344
194	ENSG00000130701	1.483335973	20.57753852	-3.7942	0.0720829	3.77E-05	0.00458
195	ENSG00000130706	897.3124454	510.533087	0.81361	1.7576039	4.35E-05	0.00511
196	ENSG00000131069	1106.300757	435.7688072	1.3441	2.5387177	1.25E-11	1.15E-08
197	ENSG00000131153	272.8944948	107.5750961	1.343	2.5367828	0.0002777	0.02181
198	ENSG00000131467	1223.421856	711.1785976	0.78264	1.7202759	0.0002154	0.01802
199	ENSG00000131470	105.9841644	45.68572772	1.214	2.3197993	0.0001967	0.01701
200	ENSG00000131473	4122.406346	2199.046556	0.90661	1.8746354	6.79E-07	0.00017
201	ENSG00000131791	629.1625287	1079.884744	-0.77937	0.5826212	0.0008147	0.04583
202	ENSG00000131910	207.9506428	437.3075399	-1.0724	0.4755273	3.15E-06	0.00062
203	ENSG00000131943	209.9351766	111.6970017	0.91035	1.8795014	0.0003825	0.0273
204	ENSG00000132002	978.1892894	540.6689645	0.85537	1.8092227	3.09E-05	0.00388
205	ENSG00000132003	13.86337619	62.93974704	-2.1827	0.2202631	6.67E-07	0.00017
206	ENSG00000132142	1050.908025	620.3093899	0.76058	1.6941716	8.31E-05	0.00891
207	ENSG00000132196	165.9606045	58.20993395	1.5115	2.8510632	9.14E-06	0.0015
208	ENSG00000132406	66.35831204	130.0559662	-0.97078	0.5102301	0.0008805	0.04858
209	ENSG00000132677	439.5576822	248.3982696	0.8234	1.7695714	0.0001514	0.01412

210	ENSG00000132692	12.87718655	45.78270384	-1.83	0.2812646	0.0004567	0.03076
211	ENSG00000132824	827.3363572	1310.787422	-0.66389	0.6311741	0.0004121	0.02869
212	ENSG00000133639	58.39991908	180.4680336	-1.6277	0.3236037	6.76E-09	3.42E-06
213	ENSG00000133935	503.596375	220.2748567	1.193	2.2862767	4.10E-08	1.65E-05
214	ENSG00000134215	86.58735408	32.30162168	1.4225	2.680496	0.0002815	0.02204
215	ENSG00000134318	1361.557281	2307.393987	-0.76101	0.5900831	2.66E-05	0.00346
216	ENSG00000134324	300.1322039	113.6055434	1.4016	2.6419442	4.46E-09	2.39E-06
217	ENSG00000134363	386.6020637	708.697611	-0.87432	0.5455109	1.32E-05	0.00198
218	ENSG00000134375	418.4680478	235.7684036	0.82775	1.7749151	0.0001494	0.01404
219	ENSG00000134684	650.1074994	1256.546114	-0.95071	0.5173778	6.25E-07	0.00016
220	ENSG00000134686	510.4348781	1106.903211	-1.1167	0.4611474	1.55E-06	0.00034
221	ENSG00000134717	211.4144611	360.0372312	-0.76807	0.5872025	0.0004432	0.0302
222	ENSG00000135049	87.65052266	172.6225473	-0.97779	0.507757	0.000215	0.01802
223	ENSG00000135069	1354.791705	2310.988603	-0.77044	0.5862387	1.74E-05	0.00249
224	ENSG00000135365	111.4041558	379.3443881	-1.7677	0.2936766	2.75E-05	0.00353
225	ENSG00000135930	531.8891496	290.7656882	0.87127	1.8292725	0.0003023	0.02302
226	ENSG00000136098	150.6057893	315.2538129	-1.0657	0.4777408	4.44E-06	0.00083
227	ENSG00000136436	282.1701112	527.8753988	-0.90363	0.5345401	1.52E-05	0.00221
228	ENSG00000136444	433.2406347	261.7754287	0.72684	1.6550101	0.0005942	0.0361
229	ENSG00000136492	197.0985053	96.95595818	1.0235	2.0328447	0.0001086	0.01119
230	ENSG00000136856	502.1616573	212.7818123	1.2388	2.3600215	9.74E-06	0.00158
231	ENSG00000137124	1445.22253	695.3308721	1.0555	2.0784384	3.45E-08	1.42E-05
232	ENSG00000137764	54.47946969	130.1070593	-1.2559	0.4187323	2.57E-05	0.00336
233	ENSG00000137876	396.1207716	700.7482017	-0.82296	0.565281	4.04E-05	0.00479
234	ENSG00000137968	23.26458989	64.90459205	-1.4802	0.3584391	0.0001419	0.01359
235	ENSG00000139269	167.2737765	372.616004	-1.1555	0.4489106	2.24E-07	7.14E-05
236	ENSG00000139428	318.3969697	140.6257477	1.179	2.2641978	4.82E-07	0.00014
237	ENSG00000139514	679.281124	1576.485594	-1.2146	0.4308925	1.35E-10	9.64E-08
238	ENSG00000139679	96.04934063	198.937868	-1.0505	0.4828008	3.53E-05	0.00433
239	ENSG00000140044	52.95156695	164.3206962	-1.6338	0.3222383	1.19E-08	5.69E-06
240	ENSG00000140297	118.4128229	48.09999068	1.2997	2.4617769	0.0007392	0.0425
241	ENSG00000140332	63.92119862	137.2511354	-1.1025	0.4657088	0.0001372	0.01329
242	ENSG00000140465	1147.309848	44.97563389	4.673	25.510159	3.34E-82	8.60E-78
243	ENSG00000140534	210.8849026	116.7775199	0.85269	1.8058649	0.0007406	0.0425
244	ENSG00000140941	240.6245494	457.1819315	-0.92598	0.5263229	1.78E-05	0.00253
245	ENSG00000140992	445.4586139	736.2287467	-0.72486	0.6050558	0.0002103	0.01788
246	ENSG00000141664	798.5140241	488.8964863	0.70779	1.6333002	0.0004076	0.02853
247	ENSG00000141956	119.3220336	59.0170039	1.0157	2.0218837	0.0007082	0.04119
248	ENSG00000143013	210.9821391	367.7381217	-0.80156	0.5737285	0.000211	0.01788
249	ENSG00000143434	22.26219416	62.48859236	-1.489	0.3562594	0.0001923	0.01674
250	ENSG00000143476	317.8309474	161.2578528	0.97889	1.9709484	3.24E-05	0.00406
251	ENSG00000143924	2969.244184	6285.913696	-1.082	0.4723735	1.98E-09	1.19E-06
252	ENSG00000143942	48.52586818	16.05036128	1.5961	3.0232494	0.0005473	0.03389
253	ENSG00000144040	392.0342098	160.6482086	1.2871	2.4403702	9.54E-09	4.73E-06
254	ENSG00000144354	636.2562778	361.5561317	0.81539	1.7597738	8.62E-05	0.00917
255	ENSG00000144485	938.3446548	348.3694514	1.4295	2.6935335	1.15E-12	1.19E-09
256	ENSG00000144749	81.62399373	153.5023958	-0.9112	0.5317426	0.0008967	0.04926
257	ENSG00000144867	522.8878457	281.5441606	0.89314	1.8572139	1.33E-05	0.00198
258	ENSG00000145050	880.5553247	331.3076476	1.4102	2.65774	1.96E-12	1.94E-09
259	ENSG00000145194	134.6606427	65.20420402	1.0463	2.0652265	0.0002415	0.01963
260	ENSG00000145217	179.7794139	84.8811699	1.0827	2.1179962	0.0004051	0.02844
261	ENSG00000145555	330.1542663	762.7275994	-1.208	0.4328683	2.68E-09	1.53E-06
262	ENSG00000145623	503.9962848	828.7899665	-0.71759	0.6081124	0.0002689	0.02126
263	ENSG00000145907	1552.452181	978.7533219	0.66553	1.5861509	0.0005039	0.03225
264	ENSG00000145920	6130.858023	2731.271538	1.1665	2.2446648	8.66E-11	6.56E-08
265	ENSG00000146083	335.1014206	733.7512336	-1.1307	0.4566941	2.39E-08	1.06E-05
266	ENSG00000146278	120.2555534	252.1642598	-1.0683	0.4768806	1.14E-05	0.00176
267	ENSG00000146376	785.2207216	1245.341646	-0.66537	0.630527	0.0003645	0.02638
268	ENSG00000146678	3545.852674	18798.25545	-2.4064	0.1886259	5.94E-07	0.00016
269	ENSG00000147155	662.475385	317.6837063	1.0603	2.0853651	3.65E-07	0.00011
270	ENSG00000147383	508.4219836	157.333373	1.6922	3.2314911	4.15E-14	5.10E-11
271	ENSG00000147526	436.5869588	976.2462845	-1.161	0.4472025	7.98E-08	2.94E-05
272	ENSG00000147852	82.62638945	177.9091748	-1.1065	0.4644194	0.0003853	0.02742
273	ENSG00000148229	434.1619999	255.3942764	0.76551	1.6999708	0.000491	0.03204
274	ENSG00000148737	138.658071	276.8671773	-0.99766	0.5008116	2.55E-05	0.00335
275	ENSG00000149150	428.2246045	694.4059854	-0.69741	0.6166783	0.0005144	0.03256
276	ENSG00000149231	267.3691637	440.2275241	-0.71942	0.6073416	0.0008236	0.04623
277	ENSG00000149485	1232.227496	740.9196237	0.73388	1.6631059	0.0001518	0.01412
278	ENSG00000149547	1344.238189	828.1587533	0.69881	1.6231654	0.00026	0.02077
279	ENSG00000149636	341.2738044	171.8683072	0.98963	1.9856757	1.21E-05	0.00186

280	ENSG00000149792	392.5799744	220.8758174	0.82975	1.7773773	0.0001706	0.01528
281	ENSG00000150347	349.4295309	705.7741533	-1.0142	0.4951028	6.10E-07	0.00016
282	ENSG00000150593	109.4763433	246.6291135	-1.1717	0.443898	2.09E-06	0.00043
283	ENSG00000150907	208.9854507	393.0878828	-0.91145	0.5316505	3.34E-05	0.00415
284	ENSG00000151012	114.4153945	866.1842634	-2.9204	0.1320906	6.36E-40	8.20E-36
285	ENSG00000151062	106.4772592	259.6555676	-1.2861	0.410058	0.0002032	0.01745
286	ENSG00000151224	749.4829065	416.4355996	0.8478	1.7997543	1.80E-05	0.00253
287	ENSG00000151276	341.0955373	554.9466963	-0.70217	0.614647	0.0005796	0.03538
288	ENSG00000151292	119.7746131	231.7893572	-0.95249	0.5167398	9.96E-05	0.01043
289	ENSG00000151632	2595.609632	922.6034724	1.4923	2.8133714	3.88E-15	5.65E-12
290	ENSG00000151693	189.1887306	383.9754883	-1.0212	0.4927064	0.0004906	0.03204
291	ENSG00000151914	827.9822189	1539.73293	-0.89501	0.5377435	0.0002519	0.02041
292	ENSG00000152409	68.81568307	205.3683768	-1.5774	0.3350852	0.0006469	0.03805
293	ENSG00000152527	25.24912372	93.5778724	-1.8899	0.2698258	0.0002007	0.01729
294	ENSG00000152782	164.3921865	62.13615053	1.4036	2.6456093	5.78E-07	0.00016
295	ENSG00000152894	738.5943053	1209.810008	-0.71193	0.6105029	0.0001708	0.01528
296	ENSG00000153250	338.6908361	555.4913537	-0.7138	0.6097121	0.0005966	0.03616
297	ENSG00000154035	14.36457405	45.73334742	-1.6707	0.3141009	0.00041	0.02863
298	ENSG00000154930	491.6081415	278.4882641	0.81989	1.7652714	0.0001416	0.01359
299	ENSG00000155111	179.1242581	335.5897794	-0.90574	0.5337589	7.50E-05	0.00816
300	ENSG00000155158	30.18007189	99.61873997	-1.7228	0.3029602	5.24E-07	0.00015
301	ENSG00000155621	31.69176852	74.00830295	-1.2236	0.4282129	0.0008703	0.04821
302	ENSG00000156253	70.84478367	147.8174435	-1.0611	0.4792665	0.0001529	0.01417
303	ENSG00000156515	0.986189633	17.65929101	-4.1624	0.0558461	7.70E-05	0.00834
304	ENSG00000156639	580.709588	977.0568279	-0.75063	0.594344	0.0001158	0.0116
305	ENSG00000156976	1417.891635	2485.596555	-0.80984	0.5704451	1.21E-05	0.00186
306	ENSG00000158042	315.9679593	177.9057013	0.82867	1.7760473	0.0002625	0.02087
307	ENSG00000158109	329.3828074	187.0639788	0.81623	1.7607987	0.0003169	0.02373
308	ENSG00000158865	36.65107735	9.961873997	1.8794	3.6792201	0.0004312	0.02978
309	ENSG00000159399	1494.537254	2450.656466	-0.71347	0.6098515	9.47E-05	0.01004
310	ENSG00000159596	137.692139	248.539392	-0.85203	0.5540047	0.0004402	0.03008
311	ENSG00000160183	8.920273453	51.46765615	-2.5285	0.1733188	8.33E-05	0.00891
312	ENSG00000160285	2168.882187	728.0620796	1.5748	2.9789419	2.83E-10	1.97E-07
313	ENSG00000160949	469.9443818	205.3822705	1.1942	2.2881791	5.82E-08	2.24E-05
314	ENSG00000160957	558.3582603	336.197687	0.73188	1.6608019	0.0006169	0.03674
315	ENSG00000161011	1551.801077	867.2276951	0.83946	1.7893803	5.50E-06	0.00095
316	ENSG00000161547	1059.93888	670.6634173	0.66032	1.5804331	0.0005008	0.03225
317	ENSG00000161682	58.41207365	132.4702292	-1.1813	0.440954	7.00E-05	0.00778
318	ENSG00000161981	306.0777023	135.2473543	1.1783	2.2630995	0.0005022	0.03225
319	ENSG00000161996	345.496927	162.2623485	1.0903	2.1291831	2.31E-06	0.00047
320	ENSG00000162769	403.7076155	188.826188	1.0963	2.1380565	1.78E-05	0.00253
321	ENSG00000163013	141.014154	245.3743623	-0.79914	0.5746917	0.0007871	0.04457
322	ENSG00000163347	775.5168347	1217.528266	-0.65073	0.6369579	0.0004332	0.02984
323	ENSG00000163918	341.132001	196.1746366	0.79819	1.7389181	0.0005029	0.03225
324	ENSG00000163950	548.1896392	320.8963556	0.77257	1.7083102	0.0001967	0.01701
325	ENSG00000164045	273.8806844	102.1897558	1.4223	2.6801245	1.00E-06	0.00025
326	ENSG00000164171	649.3076798	1330.69554	-1.0352	0.4879482	6.62E-08	2.47E-05
327	ENSG00000164211	474.4470595	199.8367039	1.2474	2.3741318	4.70E-09	2.47E-06
328	ENSG00000164284	99.04437324	185.1484902	-0.90254	0.5349441	0.0005124	0.03256
329	ENSG00000164574	302.003295	169.2531451	0.83538	1.784327	0.0002938	0.02246
330	ENSG00000164683	42.09132642	167.6355318	-1.9937	0.2510941	3.86E-05	0.00466
331	ENSG00000164749	43.58276544	99.11301871	-1.1853	0.4397331	0.000325	0.0242
332	ENSG00000165280	3150.664944	1649.677415	0.93347	1.9098641	4.03E-07	0.00012
333	ENSG00000165782	356.9839626	202.106371	0.82074	1.7663118	0.0002151	0.01802
334	ENSG00000165868	233.2240757	117.8313719	0.98499	1.9792996	5.46E-05	0.00623
335	ENSG00000165959	104.9453049	226.051575	-1.107	0.4642584	1.06E-05	0.00166
336	ENSG00000166016	184.7265682	380.5056366	-1.0425	0.4854855	1.93E-05	0.00264
337	ENSG00000166123	2199.632333	3395.088755	-0.62619	0.6478852	0.0004755	0.03171
338	ENSG00000166477	315.3533188	589.2096896	-0.90181	0.5352148	1.03E-05	0.00166
339	ENSG00000166508	2219.320853	1174.7479	0.91777	1.8891929	0.0007119	0.04129
340	ENSG00000166925	112.3822424	205.2731374	-0.86913	0.5474769	0.0005752	0.0352
341	ENSG00000166986	1028.3229	1619.436606	-0.6552	0.6349875	0.000493	0.03207
342	ENSG00000167323	417.3481579	698.3815584	-0.74276	0.597595	0.0002135	0.01802
343	ENSG00000167508	1890.071746	491.8606168	1.9421	3.8426458	1.24E-23	4.45E-20
344	ENSG00000167601	43.1018252	107.0130716	-1.312	0.4027621	4.67E-05	0.00547
345	ENSG00000167695	456.6389248	228.1255532	1.0012	2.0016642	0.0002276	0.01879
346	ENSG00000167778	204.4868245	115.7660774	0.8208	1.7663852	0.0007529	0.0431
347	ENSG00000168137	594.718819	953.5575685	-0.68111	0.6236852	0.000501	0.03225
348	ENSG00000168237	462.883045	220.4246627	1.0704	2.1000155	9.45E-07	0.00024
349	ENSG00000168297	41.59012856	116.470961	-1.4857	0.3570752	3.40E-06	0.00066

350	ENSG00000168300	57.89466969	116.8762327	-1.0135	0.4953431	0.0007132	0.04129
351	ENSG00000168439	2041.852955	1060.448622	0.9452	1.9254558	3.85E-07	0.00011
352	ENSG00000168496	667.899428	239.5534978	1.4793	2.7881342	1.36E-06	0.00031
353	ENSG00000169174	1505.638204	699.0120433	1.107	2.1539728	1.43E-06	0.00032
354	ENSG00000169710	12168.42819	3888.11583	1.646	3.1296471	7.88E-20	1.69E-16
355	ENSG00000169715	218.9000169	97.90936067	1.1608	2.2358137	5.26E-06	0.00094
356	ENSG00000169908	2410.082053	3870.330767	-0.68337	0.622709	0.0001754	0.01555
357	ENSG00000169976	626.753776	373.7789893	0.74571	1.6767993	0.0002902	0.02239
358	ENSG00000170222	9.413368269	35.51948115	-1.9158	0.2650249	0.0005156	0.03256
359	ENSG00000170271	73.75068276	209.4972292	-1.5062	0.3520373	1.59E-08	7.44E-06
360	ENSG00000170525	223.7945013	103.2418711	1.1161	2.1676022	8.94E-06	0.00148
361	ENSG00000170855	309.0160136	158.1387062	0.96649	1.9540806	0.0002852	0.0222
362	ENSG00000170915	701.7852185	1187.071935	-0.75831	0.5911885	6.28E-05	0.00709
363	ENSG00000171206	381.2509482	702.6230175	-0.88201	0.5426109	7.88E-06	0.00133
364	ENSG00000171241	309.4645417	181.8812743	0.76678	1.701468	0.0007767	0.04427
365	ENSG00000171425	89.62290192	267.1503487	-1.5757	0.3354803	0.0003543	0.02586
366	ENSG00000171793	546.3266511	281.6004639	0.95611	1.9400717	4.50E-06	0.00083
367	ENSG00000171940	503.8423269	907.4752526	-0.84889	0.5552117	1.58E-05	0.00229
368	ENSG00000171988	259.8187836	481.1851755	-0.88909	0.5399546	2.74E-05	0.00352
369	ENSG00000172115	960.9147647	497.8503912	0.9487	1.9301326	1.64E-05	0.00236
370	ENSG00000172164	748.9168843	1491.253719	-0.99365	0.5022056	1.40E-07	4.81E-05
371	ENSG00000172216	215.7874901	779.6204932	-1.8532	0.2767778	1.92E-06	0.00041
372	ENSG00000172893	3692.714611	1412.545127	1.3864	2.6142552	5.60E-14	6.55E-11
373	ENSG00000173482	524.7184217	946.0679974	-0.8504	0.5546309	1.31E-05	0.00197
374	ENSG00000173530	447.1607322	184.3925134	1.278	2.4250256	3.64E-09	1.99E-06
375	ENSG00000173692	1367.196054	652.3694397	1.0675	2.0957985	3.47E-08	1.42E-05
376	ENSG00000173812	1381.132358	3235.811097	-1.2283	0.4268201	0.0001756	0.01555
377	ENSG00000173930	79.19903488	22.74328266	1.8	3.4822023	5.34E-06	0.00094
378	ENSG00000174485	204.4341547	407.2703752	-0.99435	0.501962	0.0001883	0.01645
379	ENSG00000174744	366.0095756	175.5970981	1.0596	2.0843535	7.20E-05	0.00789
380	ENSG00000174951	2.477628652	35.72038028	-3.8497	0.0693625	4.72E-06	0.00086
381	ENSG00000175155	48.01251574	132.5213223	-1.4647	0.3623109	1.47E-06	0.00033
382	ENSG00000175197	32.13624506	141.0205991	-2.1336	0.2278885	0.0002903	0.02239
383	ENSG00000175376	157.0160219	79.04293817	0.9902	1.9864604	0.0003771	0.02714
384	ENSG00000175793	417.0495362	138.8176556	1.587	3.0042399	1.69E-08	7.76E-06
385	ENSG00000176046	141.0668238	297.2472902	-1.0753	0.4745724	3.71E-06	0.0007
386	ENSG00000176532	95.1158208	44.37640995	1.0999	2.1433984	0.0008563	0.04755
387	ENSG00000177084	659.4438887	355.4711179	0.89152	1.8551296	0.000115	0.0116
388	ENSG00000177169	167.3021371	295.2789717	-0.81962	0.5665912	0.0004375	0.02998
389	ENSG00000177508	133.1611006	302.1727922	-1.1822	0.440679	5.69E-07	0.00016
390	ENSG00000179820	539.4435813	997.0368792	-0.88617	0.5410486	4.75E-06	0.00086
391	ENSG00000179913	41.6144377	92.0200358	-1.1449	0.452221	0.0006276	0.03714
392	ENSG00000180448	150.920617	265.0972112	-0.81273	0.5693036	0.0005082	0.03241
393	ENSG00000182022	454.6057727	881.4032406	-0.95519	0.5157737	0.0006058	0.03647
394	ENSG00000182095	698.3416579	1256.903766	-0.84787	0.5556044	7.68E-06	0.0013
395	ENSG00000182162	207.4899602	93.228904	1.1542	2.2256088	6.46E-06	0.00111
396	ENSG00000182199	1548.55485	2600.656012	-0.74795	0.5954491	0.0001294	0.01268
397	ENSG00000182481	1522.497803	977.8319086	0.63878	1.5570119	0.0006627	0.0388
398	ENSG00000182541	321.7716544	532.6017385	-0.72702	0.6041505	0.0004821	0.03185
399	ENSG00000182621	324.7383264	590.1137357	-0.86172	0.5502961	2.52E-05	0.00334
400	ENSG00000182704	680.0011042	410.6960807	0.72747	1.6557329	0.0002188	0.01824
401	ENSG00000183098	292.5007932	484.207346	-0.72718	0.6040835	0.0004853	0.03198
402	ENSG00000183248	22.77554659	63.79443669	-1.4859	0.3570257	0.0002365	0.01934
403	ENSG00000183386	68.35094892	176.1893752	-1.3661	0.3879385	0.0001685	0.01521
404	ENSG00000183684	1023.056866	466.4667093	1.133	2.1931432	2.66E-06	0.00053
405	ENSG00000183856	240.0625787	406.5213453	-0.75992	0.5905291	0.0004555	0.03076
406	ENSG00000184207	383.2962549	169.4081613	1.178	2.2626289	2.54E-07	7.89E-05
407	ENSG00000184384	193.0929739	638.4942344	-1.7254	0.3024147	0.0001458	0.01383
408	ENSG00000184897	481.6733178	981.2437204	-1.0266	0.4908656	1.29E-07	4.48E-05
409	ENSG00000184990	591.0686307	349.6294127	0.7575	1.6905586	0.0002691	0.02126
410	ENSG00000185019	48.03682489	102.3837081	-1.0918	0.4691756	0.0006968	0.04062
411	ENSG00000185090	247.5157223	117.7361325	1.072	2.1023458	1.88E-05	0.00261
412	ENSG00000185112	38.13441332	185.6472646	-2.2834	0.2054131	1.24E-06	0.00029
413	ENSG00000185262	111.436568	531.2361174	-2.2531	0.2097729	1.87E-09	1.15E-06
414	ENSG00000185298	454.0559566	237.5817059	0.93445	1.9111619	1.79E-05	0.00253
415	ENSG00000185658	201.4755858	352.1830613	-0.80572	0.5720765	0.0003169	0.02373
416	ENSG00000185813	1905.134851	1035.010568	0.88025	1.8406942	3.61E-06	0.00069
417	ENSG00000186205	311.8449337	175.9478032	0.82568	1.7723702	0.0003913	0.02769
418	ENSG00000186591	220.7751596	425.7384729	-0.94739	0.5185698	1.40E-05	0.00206
419	ENSG00000187134	2972.357902	1809.550543	0.71598	1.6425986	8.28E-05	0.00891

420	ENSG00000188554	601.8437892	1704.938569	-1.5023	0.3529902	0.0001186	0.01184
421	ENSG00000189057	151.5190515	48.00301456	1.6583	3.1564437	0.0002305	0.01891
422	ENSG00000189060	2245.910318	10104.93182	-2.1697	0.2222569	3.48E-31	1.79E-27
423	ENSG00000196139	1068.127019	645.6591511	0.72624	1.6543219	0.0001361	0.01323
424	ENSG00000196421	506.6116653	255.1899038	0.98931	1.9852353	3.60E-06	0.00069
425	ENSG00000196584	159.4450322	78.0349691	1.0309	2.0432985	0.0001631	0.01479
426	ENSG00000197256	255.0296387	143.1932901	0.8327	1.7810154	0.0006175	0.03674
427	ENSG00000197451	1570.264367	891.9688205	0.81594	1.7604448	0.0001844	0.01622
428	ENSG00000197461	9.902411562	52.57433807	-2.4085	0.1883516	0.0002878	0.02233
429	ENSG00000197728	380.7902656	214.2885557	0.82944	1.7769955	0.0003494	0.02565
430	ENSG00000197785	395.2075094	217.9575699	0.85856	1.8132276	0.0001011	0.01054
431	ENSG00000197969	281.595986	479.6766953	-0.76844	0.5870519	0.0002604	0.02077
432	ENSG00000198018	157.4118801	75.52199329	1.0596	2.0843535	0.0001742	0.01553
433	ENSG00000198074	635.7319618	162.4580374	1.9684	3.9133388	2.45E-05	0.00328
434	ENSG00000198075	0.99429268	16.85395778	-4.0833	0.0589935	0.0001408	0.01358
435	ENSG00000198189	336.572602	579.5456908	-0.784	0.5807544	0.0001333	0.01301
436	ENSG00000198369	431.7816078	741.4590709	-0.78007	0.5823385	7.19E-05	0.00789
437	ENSG00000198417	72.26329526	26.76647536	1.4328	2.6997017	0.0001459	0.01383
438	ENSG00000198554	275.8125484	127.6980065	1.111	2.1599531	5.45E-06	0.00095
439	ENSG00000198796	91.562869	178.8573671	-0.96597	0.5119341	0.0002922	0.02246
440	ENSG00000198814	257.9638985	671.2591678	-1.3797	0.3842987	3.50E-11	3.11E-08
441	ENSG00000198830	994.7924522	554.1587303	0.8441	1.7951445	2.11E-05	0.00286
442	ENSG00000198839	88.64481534	223.1333275	-1.3318	0.3972723	2.15E-07	6.93E-05
443	ENSG00000198912	186.6989474	88.7528199	1.0728	2.1035119	5.17E-05	0.00597
444	ENSG00000198937	174.803899	87.14215344	1.0043	2.00597	0.0001689	0.01521
445	ENSG00000204388	627.4129833	97.00010444	2.6934	6.4683601	2.94E-32	2.52E-28
446	ENSG00000204389	292.7276786	52.8791602	2.4688	5.5358314	1.49E-20	3.84E-17
447	ENSG00000204516	229.7197421	110.8916685	1.0507	2.0715347	3.07E-05	0.00388
448	ENSG00000204520	218.9162229	92.47466392	1.2432	2.3672302	1.10E-06	0.00026
449	ENSG00000206053	1193.875491	755.9074493	0.65937	1.5793928	0.000617	0.03674
450	ENSG00000213398	144.5508997	72.75007833	0.99056	1.9869561	0.0003052	0.02313
451	ENSG00000214290	76.7173547	164.0652305	-1.0966	0.4676172	0.0001193	0.01187
452	ENSG00000221963	46.00772429	126.2371461	-1.4562	0.3644518	2.53E-05	0.00334
453	ENSG00000226137	39.08819077	84.62744091	-1.1144	0.4618832	0.0008719	0.04821
454	ENSG00000227184	348.6459175	198.5888996	0.81198	1.7556193	0.000363	0.02634
455	ENSG00000228709	98.99575496	29.48382374	1.7474	3.3575293	5.41E-07	0.00015
456	ENSG00000231312	62.40950198	157.021604	-1.3311	0.3974651	1.29E-05	0.00195
457	ENSG00000232803	3.969067672	23.24379375	-2.55	0.170755	0.0005045	0.03225
458	ENSG00000234456	89.13791015	168.1429897	-0.91558	0.5301307	0.0006113	0.03663
459	ENSG00000234899	1.479284449	16.20016726	-3.453	0.0913153	0.0005701	0.03497
460	ENSG00000235194	204.1112239	100.8258715	1.0175	2.0244079	0.0005934	0.0361
461	ENSG00000235878	18.81863349	1.459123752	3.689	12.897325	0.0001105	0.0113
462	ENSG00000236404	1.49143902	21.88511956	-3.8752	0.0681473	1.92E-05	0.00264
463	ENSG00000244062	139.624003	46.69369679	1.5802	2.990113	1.05E-05	0.00166
464	ENSG00000244405	363.9440113	729.1253435	-1.0025	0.4991343	5.99E-07	0.00016
465	ENSG00000247077	389.8118271	218.2095622	0.83706	1.786406	0.0002055	0.01753
466	ENSG00000247844	85.72676168	172.0657329	-1.0051	0.4982356	0.0004546	0.03076
467	ENSG00000253196	21.28005605	68.32335065	-1.6829	0.3114559	2.47E-05	0.00329
468	ENSG00000254054	362.4444693	591.3667501	-0.70629	0.6128942	0.0005653	0.03476
469	ENSG00000254682	36.13367339	9.609432165	1.9108	3.7601755	0.0003491	0.02565
470	ENSG00000254858	127.2966326	54.69072578	1.2188	2.3275304	5.40E-05	0.00619
471	ENSG00000256162	428.7784722	201.3608145	1.0904	2.1293307	0.0001295	0.01268
472	ENSG00000270145	28.23605328	3.723580733	2.9228	7.5831644	3.98E-05	0.00475
